# Variable Gene Dispersal Conditions and Spatial Deforestation Patterns Can Interact to Affect Tropical Tree Conservation Outcomes

**DOI:** 10.1371/journal.pone.0127745

**Published:** 2015-05-22

**Authors:** Yamini Kashimshetty, Stephan Pelikan, Steven H. Rogstad

**Affiliations:** 1 Department of Biological Sciences, University of Cincinnati, Cincinnati, OH, United States of America; 2 Department of Mathematical Sciences. University of Cincinnati, Cincinnati, OH, United States of America; Chinese Academy of Sciences, CHINA

## Abstract

Tropical lowland rain forest (TLRF) biodiversity is under threat from anthropogenic factors including deforestation which creates forest fragments of different sizes that can further undergo various internal patterns of logging. Such interventions can modify previous equilibrium abundance and spatial distribution patterns of offspring recruitment and/or pollen dispersal. Little is known about how these aspects of deforestation and fragmentation might synergistically affect TLRF tree recovery demographics and population genetics in newly formed forest fragments. To investigate these TLRF anthropogenic disturbance processes we used the computer program NEWGARDEN (NG), which models spatially-explicit, individual-based plant populations, to simulate 10% deforestation in six different spatial logging patterns for the plant functional type of a long-lived TLRF canopy tree species. Further, each logging pattern was analyzed under nine varying patterns of offspring versus pollen dispersal distances that could have arisen post-fragmentation. Results indicated that gene dispersal condition (especially via offspring) had a greater effect on population growth and genetic diversity retention (explaining 98.5% and 88.8% of the variance respectively) than spatial logging pattern (0.2% and 4.7% respectively), with ‘Near’ distance dispersal maximizing population growth and genetic diversity relative to distant dispersal. Within logged regions of the fragment, deforestation patterns closer to fragment borders more often exhibited lower population recovery rates and founding genetic diversity retention relative to more centrally located logging. These results suggest newly isolated fragments have populations that are more sensitive to the way in which their offspring and pollen dispersers are affected than the spatial pattern in which subsequent logging occurs, and that large variation in the recovery rates of different TLRF tree species attributable to altered gene dispersal regimens will be a likely outcome of fragmentation. Conservation implications include possible manual interventions (manual manipulations of offspring dispersers and/or pollinators) in forest fragments to increase population recovery and genetic diversity retention.

## Introduction

Tropical lowland rain forests (TLRF’s) are high-diversity biomes that continue to be under threat from anthropogenic deforestation for resource extraction and land-use change, resulting in the breakup of once continuous forest into fragments of various sizes [1, 2, 3, 4, 5]. These forest remnants can range from 3 ha to over 120,000 ha [6, 7]. A large majority of tropical tree species exist at low population densities making them highly susceptible to local habitat disruption [8, 9]. Conservation of these vulnerable biomes is critical because they are not only reservoirs of biodiversity, but TLRF’s provide important ecosystem services such as moderating biogeochemical cycles and the provisioning of forest architecture, microclimate, and nutritional resources for other organisms [10, 11, 12, 9].

It has been suggested that a large number of low-density tropical tree species are highly outcrossed, thus highlighting the importance of gene flow in these populations which can in turn affect the spatial distribution of individuals, rates of population growth, and population genetics through space and time [13, 14]. Plant gene dispersal has been found to commonly follow a leptokurtic distribution, involving occasional long-distance dispersal coupled with mainly short-distance dispersal ranges [15, 16]. Fragmentation can affect these naturally-occurring gene dispersal curves (kernels) of TLRF tree populations by bringing about changes in the natural abundance and/or behavior of offspring and/or pollen dispersal vectors [17, 18]. For example, certain pollinators (e.g. some species of large bees and hummingbirds) disperse pollen to greater distances following fragmentation by readily crossing resource-poor areas between fragments that are separated by a few hundred meters to several hundred kilometers, respectively [19, 17]. In contrast, some dispersers in TLRF’s (e.g. certain species of bees, birds and small beetles) are less likely to cross open pasture beyond fragment area, thereby restricting gene dispersal distance to within the fragment [20, 21]. Obviously, not all gene dispersal vectors will respond in the same way to fragmentation, and post-fragmentation alterations in gene dispersal cannot be generalized for all species or situations [22, 23]. As such, the effects of a range of post-fragmentation alterations in offspring and pollen dispersal distances need to be tested to determine how fragmentation might affect population growth and genetic diversity trends of TLRF tree populations.

TLRF fragments created as a result of initial deforestation can undergo further disturbance in the form of different types of logging activities from clear cutting to selective logging and Reduced Intensity Logging (RIL) [24, 25, 26]. Clear-cutting is the removal of essentially all above-ground biomass, whereas selective logging is a less intense form of deforestation that involves the removal of only the economically valuable trees, these usually being the largest trees [27, 28, 29]. Studies on selective logging in Amazonian forests showed that logging fewer, larger tracts of TLRF’s was advantageous in reducing any negative impacts on the functional composition of the remaining stand (e.g., increasing the number of early successional species) [30]. The life-history characteristics of the tree species being selectively-logged, e.g. minimum reproduction DBH (cm), has also been reported as being an important factor in determining the overall sustainability of population genetic diversity from this type of deforestation [31].

Reduced Intensity Logging (RIL) is a lower-intensity form of selective logging where less-damaging wood transportation systems are also coupled with measures implemented to better protect pre- and post-logging seedlings [32, 25, 33]. Previous RIL simulation studies have shown that certain life-history traits are important determinants in post-logging population recovery, with size-class distributions being the most influential factor, followed by growth rates (DBH increments) and mortality rates [34]. The success of RIL was found to partly depend upon sufficiently long cutting cycles that enabled natural regeneration processes to occur [24].

One aspect of logging that has not been fully explored is whether different spatial logging patterns of TLRF trees in forest fragments might affect the demographic dynamics and the genetics of populations with varying life-history characteristics. For example, are there certain geometrical patterns of tree removal which are more sustainable in terms of improved population re-growth and genetic diversity retention over others? How do these variable patterns of logging interact with post-fragmentation alterations in life-history traits such as gene dispersal distances in terms of population recovery and genetic diversity retention? Detailed research into such issues is necessary to help reduce the high rates of rain forest biodiversity alteration and loss, [35, 36, 37]. For example, such interventions could affect existent population abundance and growth dynamics, as well as the spatial distribution patterns of offspring recruitment and/or pollen dispersal, and thus have a potential effect on the viability of the population in the short and/or long term [38].

To study these aspects of TLRF deforestation and fragmentation, the goal of this research was to investigate the effects of different spatial logging patterns on a plant functional type (that of a long-lived, tropical canopy tree population) that is commercially exploited, under a range of different offspring and pollen dispersal distances potentially resulting from post-fragmentation shifts in dispersal vector abundance and/or behavior [39, 40, 41]]. Alternatively, the experimental virtual populations examined here could be thought of as different species that share most of their life history characteristics except for offspring and pollen dispersal kernels: how would fragmented populations of these different species respond to different deforestation practices? A central objective of this study was to determine whether tree removal patterns could be found that differentially maximized population recovery and genetic diversity retention, and/or kept population subdivision and inbreeding levels low, in order to recommend more sustainable extraction practices and effective conservation strategies under particular gene dispersal scenarios. We hypothesized that TRLF tree populations would respond differently to post-fragmentation alterations in gene dispersal and variable spatial patterns and types of logging (i.e., in lines, at edges, or selective logging) in terms of their population recovery, genetic diversity retention, inbreeding, and population subdivision.

Given the stochastic nature of several processes driving population development in natural populations, a high level of experimental replication is crucial for providing statistically accurate short- and long-term insights regarding the issues we are addressing [42, 43]. Sufficient experimental replication in the field in tropical low land rain forests (TLRF’s) is currently not feasible due to expenditures and spans of time needed [34]. As such, we used a spatially-explicit computer simulation program, NEWGARDEN, which models the population growth and genetic diversity dynamics of plant populations through time, and which is also capable of highly replicated computer modeling providing statistical robustness [44], to conduct an entirely theoretical modeling study exploring how, for a ‘typical’ TLRF long-lived canopy tree, alternative deforestation methodologies in TLFR fragments can interact with differing gene dispersal patterns to affect subsequent population recovery and genetics [45, 46, 47, 48, 49].

## Methods

### NEWGARDEN

NEWGARDEN (NG) is a spatially explicit, individual-based computer program that simulates natural population development of plant species through bouts of mating, and provides output to assess population growth, genetic diversity, population subdivision and/or inbreeding level trajectories of the developing, comparative, virtual populations. Population development is conditioned by user defined input parameters such as genetic diversity of the source population; geometric number, age distribution and pattern of the founders; gene dispersal distance kernels; etc.

NG operates based on a set of user-specified input conditions that specify: 1) the characteristics of the preserve fragment system; 2) the number, geometric placement, genetic diversity, and age distribution of the founding population; and 3) several life-history characteristics such as age-specific offspring and pollen production, mortality and life-span describing the species being modeled. Each set of user-specified input conditions constitutes a “trial”. For each set of trial input conditions, the user can define the number of replicate “run” analyses using those conditions to be performed, thus allowing for statistical comparisons of mean output values for intra-trial runs (given with standard deviations) between contrasting trials. Output statistics compared across trials from each successive bout of reproduction for the entire population include means for population size, measures of genetic diversity including the number of founding alleles retained, observed and expected heterozygosity, and F_it_ values reflecting population subdivision and/or inbreeding (interpreted according to [50]). These mean values can be compared across trials using ANOVA’s and T-tests of means. In this way, output from trials differing in input conditions can be compared to examine the effect of varying one or more characteristic(s) on the population growth and genetic diversity of resultant populations [46, 48, 49].

Review of previous forest simulation models indicates NG as not fitting any one model classification (e.g., it is not entirely a physiologically-based process model, gap model or an empirical stand model; [51, 52, 53]). While NG does share some features with other programs, (e.g. it seeks to describe population growth through the processes of recruitment, growth and mortality and it can model gap and non-gap situations (see [51, 52, 53]), it is unlike any other program currently available as it combines different features of a range of models and contains unique features as well. For example, 1) it models a full range of life-history traits set by age-specific probabilities including reproduction, pollen production and mortality; 2) it is driven by explicit ‘virtual’ life-history events such as reproduction, gene dispersal and mortality which are set by age-specific parameters and not by mathematical growth equations nor any physiologically-based equations regarding photosynthesis, carbon balance and other abiotic conditions; 3) it has the ability to model different mating systems including agamospermy, different levels of selfing, and dioecy; 4) it allows the user to specify, for a given NG trial, the genetic diversity of the source population, spatial location and age structure of the founders; 5) it can model complex genetic histories including multi-locus founder genetic diversity levels where the user can set desired allele frequencies, self-incompatibility alleles and effects of inbreeding depression; 6) it models reproduction, dispersal and immigration in a spatially-explicit manner including offspring and pollen dispersal distance categorical probabilities, allowing mating events only between eligible pollen donors and pistillate individuals; 7) it can model spatially heterogeneous landscapes that can change through time; 8) it is capable of high levels of replication of comparative simulations, and calculates means and standard deviations for important measures to inform conservation practices, such as population growth, genetic diversity levels and population genetic statistics like F_it_ and F_st_ over time; and 9) output reported after each new bout of mating include both entire standing crop versus only the most recent cohort data, and includes coordinates, parentage, allelic status, and age of death of all individuals as well as other statistics if requested (S1 Table; [45, 46, 47, 48, 49]). NG is freely available, with associated materials and program updates, by agreement with Science Publishers (Enfield, NH) at: http://math.uc.edu/~pelikan/NEWGARDEN/.

### Control Equilibrium Population

Our modeling was centered on the plant functional type of a long-lived, canopy tree species since this group is commonly harvested for timber in TLRF’s [54, 39, 32, 41]. A “control equilibrium population” (one not changing in size through bouts of mating; [55] for this plant functional type was developed to enable the simulation of comparative trials of an initially stable baseline tree population that then undergoes varying spatial logging patterns and modification of gene dispersal conditions.

To develop and model this control population of a ‘typical’ TLRF long-lived canopy tree, we incorporated some of the initial input life history characteristics from one of the better studied TLRF canopy trees, *Symphonia globulifera* L. f. (Clusiaceae), a widespread species distributed in several regions of the Neotropics and the West African tropics [56, 57]. Since no single tropical tree species has been studied for all the parameters utilizable with NG (including *S*. *globulifera*), we used empirical studies on other species, or groups of species [58] thought to be typical of this plant functional type to derive other life-history parameters for these computer simulations. For example, studies on the commonly exploited canopy tree species *S*. *globulifera* provided estimates for population density, age-class distribution, mating system and preserve characteristics, while data from a 50-ha rain forest plot in Pasoh, Malaysia provided estimates for size-class distribution for the smaller size-classes, and data on mortality rates were derived from a study on tropical canopy tree species from La Selva, Costa Rica [58, 59, 60, 41]. The equilibrium population was based on our best estimates of the life history conditions for a tropical, long-lived canopy tree species and setting the reproductive value (r, average offspring produced per individual in a given round of mating) such that the population size remained stable. These control equilibrium population conditions were subsequently perturbed by altering logging patterns and gene dispersal conditions in comparative trials to analyze the resulting population growth and genetic diversity consequences of such alterations. It is important to note that the aim of this research was not to model one particular TLRF plant species. Rather, we attempted to model responses of different populations of a more or less “representative species” of a particular functional group that varied in internal patterns of culling and/or gene dispersal distances.

With a complex study like ours which explored the effects of both spatial logging pattern and gene dispersal distances (via both offspring and pollen), it was simpler to begin with a basic equilibrium population to determine the general effects of these variables separately, as well their synergistic effects. As we were mainly varying two variables in this study (spatial logging pattern, and gene dispersal condition) to specifically analyze their population growth and genetic diversity consequences, it was necessary to control for all the other parameters including the equilibrium state of the founding population in order to detect and measure the effects of the variables under scrutiny.

### Parameters held constant among trials

#### Preserve characteristics

**Fig 1:**
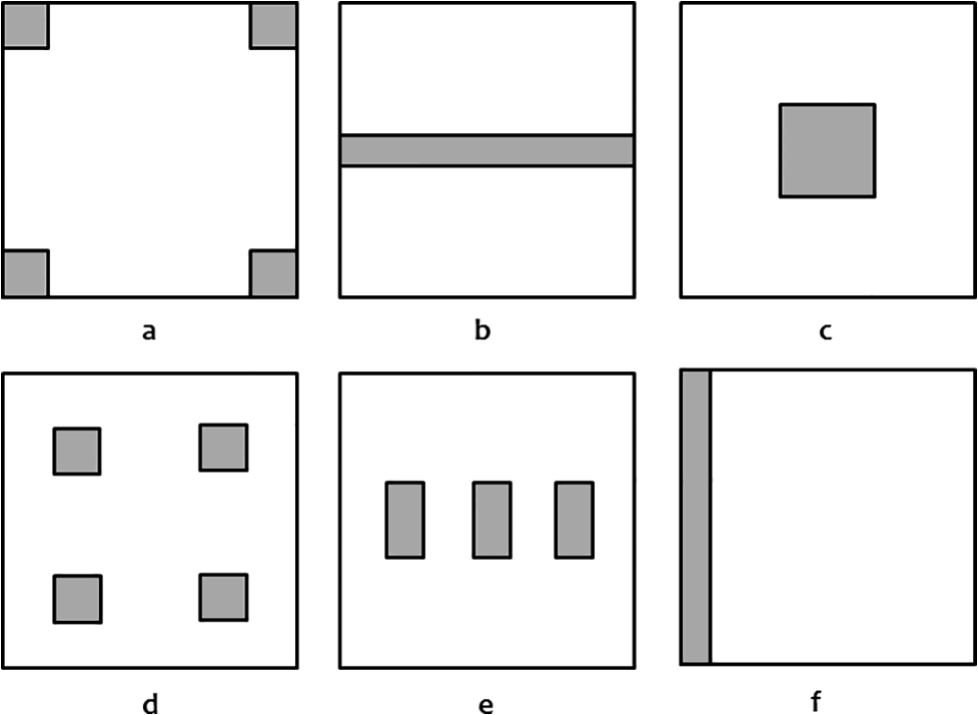
Spatial logging patterns showing 10% total deforestation of a 500 ha fragment (in grey). Fragment is 2.23 km or 160 grid points on a side. Diagram is approximately to scale. a = Corners, b = Road, c = Central Square, d = 4Squares, e = 3Strips, f = Strip. Additionally, selective logging was also performed, representing a 10% removal of only those individuals belonging to the highest age classes.

In NG simulations of the preserve system, individuals establish on a Cartesian grid system, the establishment points of which are called grid points. The distance between two grid points is meant to reflect the naturally-occurring average minimum distance between two mature, reproductive individuals in the population. We modeled a 500 ha plot based on a study of *S*. *globulifera* in the Tapajos National Forest, Brazil (equivalent to a 160 x 160 grid point fragment in NG; modified to be in the shape of a square to begin simulations with the simplest shape possible; Fig 1) with the average minimum distance between two reproductive individuals being 14m, as reported in the literature [41]. The initial control equilibrium population comprised of 965 founders randomly placed in the 500 ha un-logged fragment, with a resulting density of 1.932 individuals/ha (with ~ 0.332 reproductive individuals/ha), with equal probabilities of dispersing offspring to or receiving pollen from each distance frame respectively, relative to a pistillate individual selected for mating (see below for gene dispersal descriptions).

#### Age class distribution

The age-class distribution was based on field data of *S*. *globulifera* from Tapajos National forest Brazil [41]] and size-class distribution data and growth rates reported from Pasoh, Malaysia (further details in S1 Appendix; [58, 61]). It was set as follows: Ages 1 to 168 (1 to 15cm DBH) represent seedlings, saplings and young juveniles which collectively make up approximately 81% of the population (some tropical trees have been reported to take 98–183 years to reach 10 cm DBH; [62]), ages 169 to 236 (16 to 30 cm DBH) reflect the juvenile age classes which form ~ 10%, and greater than age 236 (30cm DBH) represent mature reproductive individuals that make up 9% of the population.

#### Loci and allelic variation

NEWGARDEN is capable of modeling founding populations with a user designated numbers of loci, each locus having a user specified number of alleles, and with each allele having a user specified initial founding population allele frequency [45, 47]. In our analyses here (and previously: e.g., [45, 46, 47, 48]) all individuals were simulated to have 30 loci, with each locus in the source population having 100 alleles, each of these alleles occurring at a frequency of 0.01. We use this loci configuration for several reasons. Although natural populations will have much more complex loci panel structures, it would not only be arbitrary to choose one at random, but we also want to use a simplified, easy-to-interpret null model for ready comparison among trials. Such low frequency alleles are a more sensitive and readily interpretable indicator of changes in population genetic diversity compared to the much less sensitive changes in heterozygosity or gene frequencies at loci with fewer, more common alleles [63]. In small populations such as found in tropical rain forests, considering the thousands of intraspecific loci, surely a considerable portion of these loci will include low frequency alleles such as those we model. Rare alleles can arise in a number of ways (e.g., genetic drift, population immigration or emigration; selection, de novo mutation, etc.). Such alleles are of especial concern in conservation efforts since they are easily lost and yet may have potential significance under differing circumstances (e.g., [64, 65, 66]) In conservation efforts, loss of the most common alleles is relatively unlikely, and thus less worrisome than loss of rare alleles. Further, we have shown that populations experiencing higher rates of loss of rare alleles tend to have a greater variance in the frequencies of more common alleles ([45], work in progress). Monitoring the magnitude of rare allele loss over time can be used as an indirect way to estimate the degree of expected variance in the frequencies of more common alleles in the population. Thus our results with loss of rare alleles reflect the expected degree of variance of common alleles under the different experimental conditions compared. Greater loss of rare alleles and the associated greater variance in the frequencies of common alleles can make populations less tractable to the processes of natural selection, and more susceptible to random genetic drift. By analyzing the rate at which rare alleles are lost, the effects of genetic drift versus natural selection can perhaps be more readily interpreted ([45], work in progress).

#### Mating system

The plant functional type modeled was a hermaphroditic species with a selfing rate of 0.098 in all trials [67, 41, 59].

#### Offspring and pollen production

In NG simulations, the user designates an age-specific reproduction rate, based on which offspring are generated from the mating of randomly selected eligible individuals. Offspring produced from mating events are distributed across eligible reproducing individuals following a Poisson distribution. The reproduction rate is meant to simulate naturally occurring phenomenon where not all the seedlings produced from a mating event will successfully establish. Studies have found fecundity to be associated with increase in tree size in certain tropical tree species, where canopy trees reach reproductive capacity at approximately 30cm DBH, this generally being the time taken to reach the canopy [62]. The age-specific reproduction rate for the control equilibrium population was as follows: Ages 140 to 167 = 0.5, 168 to 195 = 2.5, 196 to 235 = 7.8, 236 to 424 = 8.0, 425 = 0.0 (S2 Table). Pollen production pattern was similar to age-specific reproduction: Ages 140 to 167 = 0.10, 168 to 195 = 0.15, 196 to 215 = 0.20, 216 to 235 = 0.60, 236 to 424 = 1.0, 425 = 0.75, 426 = 0.0 (S2 Table).

#### Mortality

Age-specific mortality rates were based on a modified version of tropical tree mortality patterns reported from studies in La Selva, Costa Rica [60]. Mortality rates, which along with age-specific reproduction, were altered to obtain as near an equilibrium population as possible, were set as follows: Ages 0 to 10 = 25%, 11 to 25 = 14%, 26 to 50: 0.75%, 51 to 75: 0.85%, 76 to 100: 0.95%, 101 to 425: 1% (S3 Table). Further details regarding the functioning of the age-specific mortality parameter in NG, can be found in S1 Appendix.

### Parameters that varied among comparative trials

#### Offspring and pollen dispersal

In NG, offspring and pollen dispersal distance kernels were simulated via a series of hierarchically nested limiting distance frames, for each of which probabilities of dispersing offspring to, and receiving pollen from, can be designated relative to each pistillate individual selected for mating. The lower and upper limits of each distance frame are specified on the Cartesian fragment grid system [45, 46]. Offspring and pollen dispersal was varied across comparative trials to simulate ‘Near’, ‘Equal’ or ‘Far’ distances. To model these dispersal conditions, we created five distance frames, whose dimensions in the x or y direction around a pistillate individual selected for mating are (gp = grid points): a) 0 to 32 gp (448 m); b) 32 to 64 gp (896 m); c) 64 to 96 gp (1344 m); d) 96 to 128 (1792 m); and e) 128 to 160 (2240 m). ‘Near’ gene dispersal condition corresponded to 80% probability of dispersing offspring to or receiving pollen from the nearest distance frame, the ‘Equal’ condition to equal probability of dispersing offspring or receiving pollen (20%) from each distance frame, and the ‘Far’ condition to 80% probability of dispersing offspring to or receiving pollen from the furthest distance frame (S1 Fig and S4 Table).

All possible combinations of ‘Near’, ‘Equal’ and ‘Far’ variations for offspring versus pollen dispersal distances resulted in a total of nine gene dispersal regimes being tested: NONP, NPEO, FONP, NOEP, EOEP, FOEP, NOFP, EOFP, FOFP; (N = ‘Near’, F = ‘Far’, E = ‘Equal’, O = offspring, P = Pollen).

### Comparative logging patterns

The initial control equilibrium population was modified in comparative trials to study six contrasting spatial logging patterns, each comprising a total of 10% (50 ha) removal of the original 500 ha fragment in varying patterns including: one side strip (*Strip*); three equal area, vertical, equidistantly separated strips (*3Strips*); four internal, equidistantly separated, equal area subdivided squares (*4Squares*); four equal area corner squares (*Corners*); one horizontal central road (*Road*); and one central square (*CentralSq*) (Fig 1). In addition, selective logging (*Selective Logging*) was also modeled as the removal of the top 10% of individuals based on age class. All logging patterns, including an un-logged control fragment were tested with all of the nine gene dispersal conditions, resulting in a total of 72 trials being performed.

### Data Analyses

Each set of trial conditions was analyzed through 600 bouts of mating for 100 replicate runs. Replicate run output values for two different classes of summary data, ‘entire fragment’ data which included data calculated from the entire 500 ha fragment and ‘recovery region’ data calculated from only the combined logged areas of the fragment, were generated for each trial. Two-factor ANOVA’s were performed to assess the amount of variance explained by logging pattern, gene dispersal condition, and their interaction, in the output (mean population size, founding allele retention, and population subdivision/inbreeding levels). A separate set of two-factor ANOVA’s were also used to determine the relative impact of offspring versus pollen dispersal distance, and their interaction, on the amount of variance explained in the output. For this last set of ANOVA’s the mean variance reported in the results for each output was an average across all spatial logging patterns. Unpaired T-tests (Bonferroni corrected) were conducted between trials to ascertain whether means were significantly different (p<0.05).

## Results

Below are the results from two different classes of summary data: ‘entire fragment’ data which included data calculated from the entire 500 ha fragment, and ‘recovery region’ data calculated from only the combined logged areas of the fragment.

### Mean Population Size—Entire fragment

**Fig 2:**
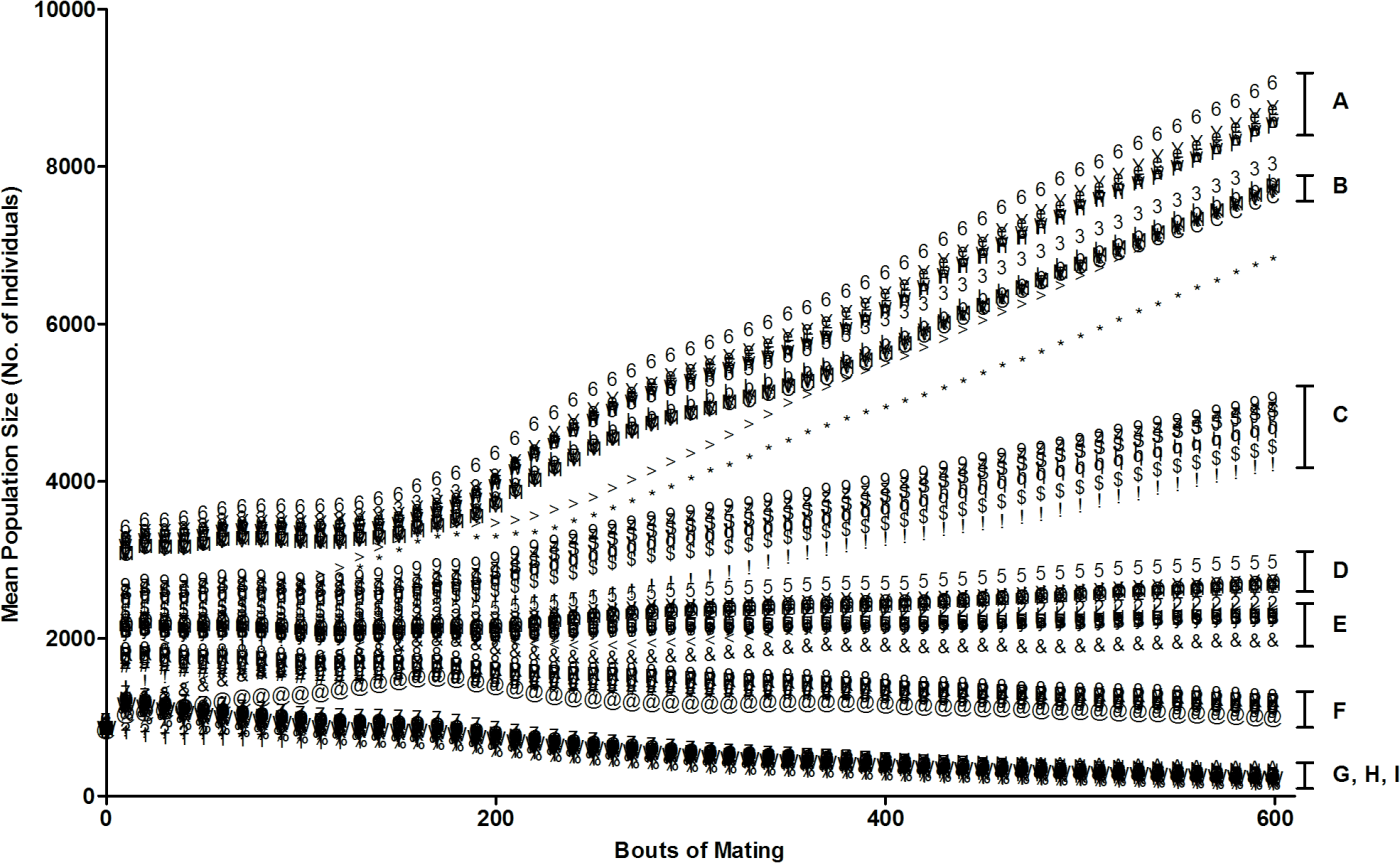
Mean population growth in the entire fragment across 600 generations. Trials varied in gene dispersal distance (offspring and pollen) and spatial logging patterns. Groups labelled A-I indicate trials grouped according to gene dispersal condition, with similar growth trajectories and endpoints. Labelled in group-descending order of mean population size after 600 bouts of mating: Gene dispersal condition is abbreviated as follows: ‘N’ = Near, ‘E’ = Equal, ‘F’ = Far, ‘P’ = Pollen, ‘O’ = Offspring, ‘Equal’, ‘Near’ and ‘Far’ refer to the probability of offspring and/or pollen being dispersed to or being received from a particular distance frame relative to a pistillate individual (for more detail see S1 Fig and S4 Table); A = NONP; B = NOEP; C = NOFP; D = EONP; E = EOEP; F = EOFP; G, H, I = FONP, FOEP, FOFP. For a complete list of logging pattern and gene dispersal condition for each trial above, see S1 Appendix.

**Fig 3:**
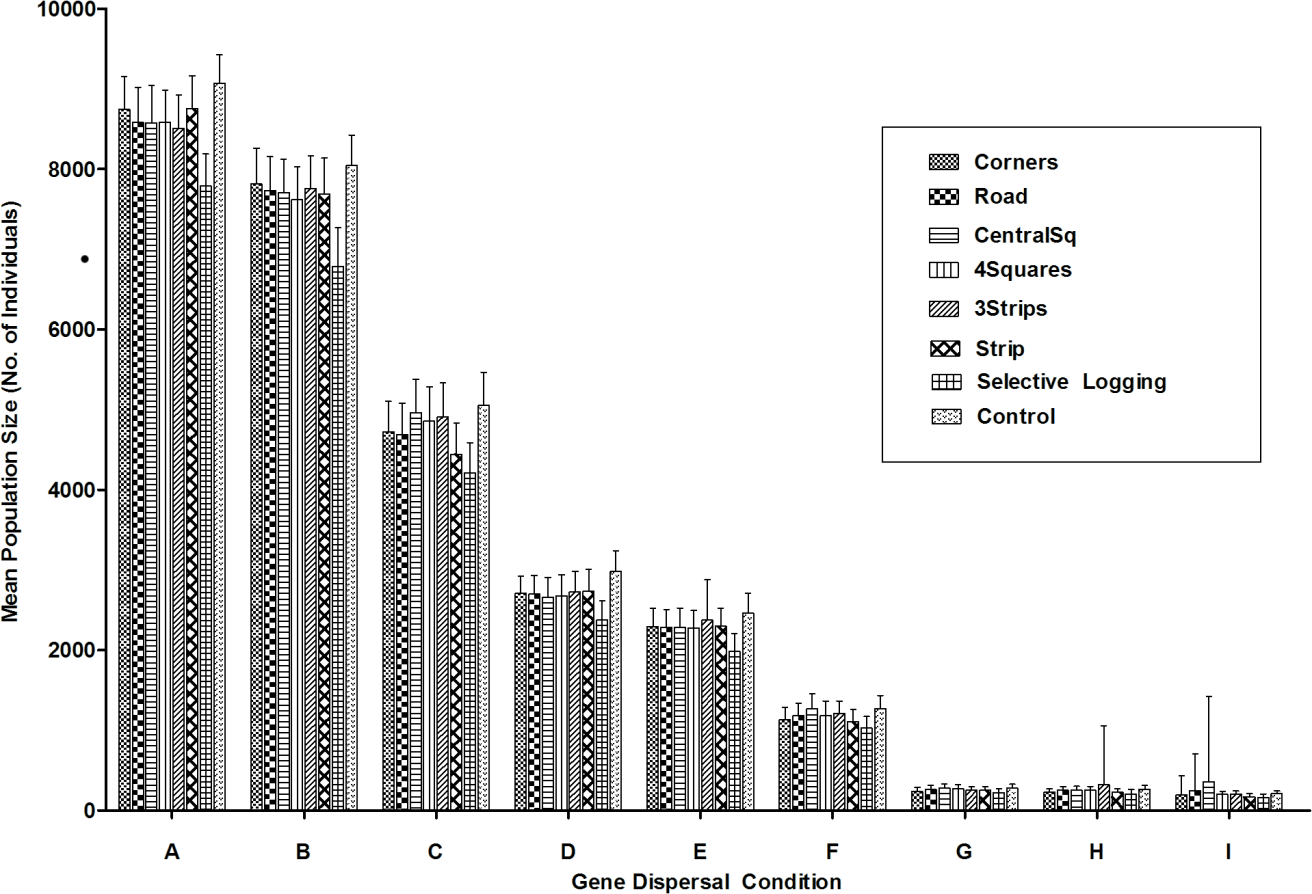
Mean population size for the entire fragment after 600 bouts of mating. Trials varied in spatial logging patterns and gene dispersal distance (offspring and pollen). Groups labelled A-I indicate trials grouped according to gene dispersal condition in descending order of mean population size. Gene dispersal condition is abbreviated as follows: ‘N’ = Near, ‘E’ = Equal, ‘F’ = Far, ‘P’ = Pollen, ‘O’ = Offspring, ‘Equal’, ‘Near’ and ‘Far’ refer to the probability of offspring and/or pollen being dispersed to, or being received from, a particular distance frame relative to a pistillate individual (for more detail see S1 Fig and S4 Table). A = NONP; B = NOEP; C = NOFP; D = EONP; E = EOEP; F = EOFP; G = FONP; H = FOEP; I = FOFP. “Control” refers to un-logged fragment, and “Selective Logging” refers to 10% removal of only those individuals belonging to the highest age-classes.

Trials with the same gene dispersal distance conditions but variable spatial logging pattern showed similar population growth through 600 bouts of mating, although there was extensive variation among trials grouped according to dispersal conditions (Fig 2). The gene dispersal condition that maximized population growth was when offspring and pollen were dispersed to/from ‘Near’ distances (Group A; Figs 2 and 3). Trials with offspring dispersal biased to ‘Near’ distances (Groups A-C) had the highest mean population sizes followed by ‘Equal’ (Groups D-F) and then ‘Far’ (Groups G-I) distances respectively, regardless of pollen dispersal condition. However, within the offspring dispersal “Near” category trials (Groups A-C), as well as the “Equal” category (Groups D-F), population growth is highest when pollen dispersal is “Near” (Groups A and D), and lowest when pollen dispersal is “Far” (Groups C and F; Figs 2 and 3). Similarly, control trials simulating unlogged fragments responded significantly to variation in gene dispersal condition (Figs 2 and 3). Two-way ANOVA analysis showed that offspring dispersal distance had a greater impact on population growth, accounting for a mean variance of 87.6%, compared to pollen dispersal (6.3%) and their interaction (4.9%).

Those trials sharing the same spatial logging pattern but with variable gene dispersal conditions did not exhibit similarities in mean population size trajectories and endpoints (Groups A-I; Fig 2). A two-way ANOVA to show the relative effects of gene dispersal condition versus logging pattern on population growth, indicated that gene dispersal condition explained higher variance in this output (98.5%, p<0.0001, F = 79781.62, df = 8, N = 9) relative to logging pattern (0.2%, p<0.0001, F = 203.20, df = 7, N = 8) and the interaction of the two (0.2%, p<0.0001, F = 23.76, df = 56, N = 72; Fig 3).00200020Selective logging tended to have the lowest population growth for the six highest population-maximizing trials (Groups A-F; Groups G-I did not manifest biologically meaningful differences; Fig 3).

### Mean Population Size—Recovery Regions

**Fig 4:**
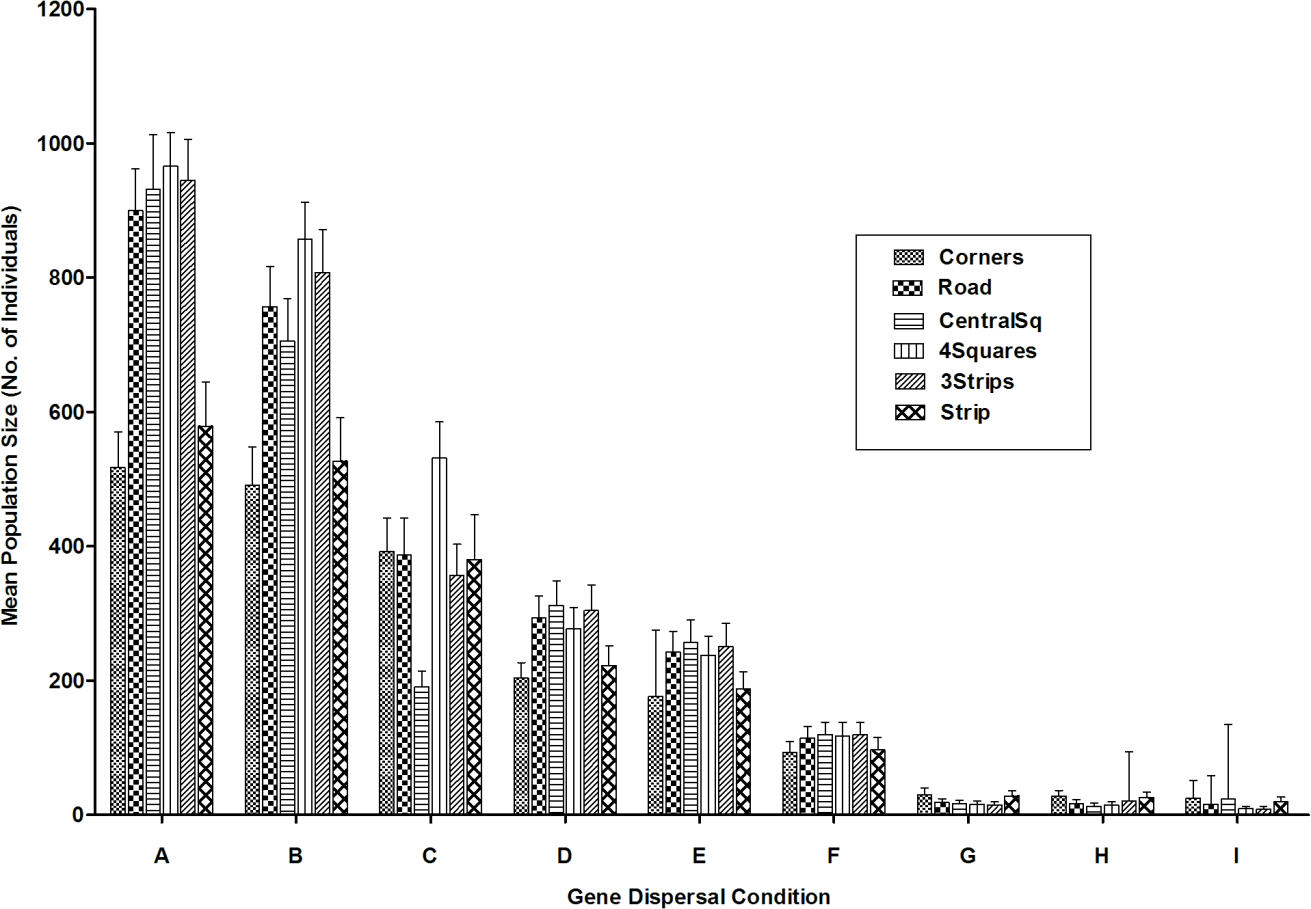
Mean population size after 600 bouts of mating for recovery regions (all logged areas combined). Trials varied in spatial logging patterns and gene dispersal conditions (offspring and pollen). Groups labelled A-I indicate trials grouped according to gene dispersal condition in descending order of mean population size: Gene dispersal condition is abbreviated as follows: ‘N’ = Near, ‘E’ = Equal, ‘F’ = Far, ‘P’ = Pollen, ‘O’ = Offspring, ‘Equal’, ‘Near’ and ‘Far’ refer to the probability of offspring and/or pollen being dispersed to or being received from a particular distance frame relative to a pistillate individual (for more detail see S1 Fig and S4 Table) A = NONP; B = NOEP; C = NOFP; D = EONP; E = EOEP; F = EOFP; G = FONP; H = FOEP; I = FOFP. Control” refers to un-logged fragment, and “Selective Logging” refers to 10% removal of only those individuals belonging to the highest age classes.

When fragment recovery regions only (combined logged areas only) were considered, trials with the same gene dispersal conditions did show some similarity in population growth trajectories and endpoints, although variation of endpoints among deforestation patterns was evident within gene dispersal groups (S2 Fig and Fig 4). A two-way ANOVA for showing the effect of offspring versus pollen dispersal distance on population growth in the recovery regions indicated that offspring dispersal distance had a greater impact, accounting for 80.9% of the mean variance relative to pollen dispersal (8.8%), and the interaction of the two dispersal types (7.4%).

Within gene dispersal groups, the recovery regions showed more variability in population recovery with regards to spatial logging pattern than did similar comparisons for the entire fragment data. In five of the nine gene dispersal condition groups, situating logged areas closer to the fragment borders (*Strip*, *Corners*) generated significantly lower mean population recovery compared to those trials that were logged further away from fragment borders (*CentralSq*, *4Squares*, *3Strips*; Groups A, B, D, E and F, Fig 4). However, the *CentralSq* extraction pattern trial with ‘Near’ offspring and ‘Far’ pollen dispersal (within Group C; Fig 4), had significantly lower levels of population recovery compared to other trials with the same gene dispersal condition. Group C was the only group in which this deforestation method exhibited such a pattern.

A two-way ANOVA showed that gene dispersal condition accounted for the most variance among recovery region endpoints (89.27%, p<0.0001, F = 26559.55, df = 8, N = 9). In the recovery regions, logging pattern, and the interaction of logging pattern and gene dispersal condition, accounted for 2.22% (p<0.0001, F = 1054.89, df = 5, N = 6) and 6.27% (p<0.0001, F = 373.03, df = 8, N = 9; Fig 4) of the variance respectively, this being more than that accounted for by these factors in the entire fragment. Thus, logging pattern, and the interaction of logging pattern and gene dispersal condition, caused greater variability in population growth in the recovery regions than in the entire fragment.

### Founding allele retention—Entire fragment

Variation in alleles retained within the entire fragment appears to be influenced by gene dispersal groupings, showing a difference of 11.0% between the most extreme trials (*Corners—*‘Near’ offspring and pollen vs. *Selective Logging—*‘Far’ offspring and ‘Equal’ pollen; S3 Fig). Within gene dispersal groups (Groups A-I, S3 Fig), S*elective Logging* trials consistently displayed significantly lower levels of founding allele retention relative to other logging patterns. Disregarding these *Selective Logging* trials, within-group variability among all other logging patterns did not show greater than a 4.8% difference in founding alleles retained (S3 Fig).

A two-way ANOVA to depict relative contributions of gene dispersal condition versus spatial logging pattern in explaining variation in founding allele retention, indicated that gene dispersal condition accounted for the highest amount of variance (88.3%, p<0.0001, F = 15387.98, df = 8, N = 9) than spatial logging pattern (4.8%, p<0.0001, F = 951.57, df = 7, N = 8) or the interaction of the two (1.9%, p<0.0001, F = 46.55, df = 56, N = 72; S3 Fig). As expected, those trials and gene dispersal conditions that maximized population growth (‘Near’ followed by ‘Equal’ then ‘Far’ offspring dispersal) generally retained the highest number of founding alleles (see S1 Appendix; S3 Fig). Variation in offspring dispersal distance had a greater impact on alleles retained, as a two-way ANOVA indicated that offspring dispersal explained a mean variance of 90.3%, compared to pollen dispersal (3.7%) and the interaction of the two dispersal types (0.43%). Control trials responded significantly to variation in offspring dispersal, with ‘Near’ dispersal (Groups A-C) retaining the most alleles, followed by ‘Equal’ and ‘Far’ respectively (Groups D-F & G-I respectively; S3 Fig).

### Founding allele retention—Recovery region

**Fig 5:**
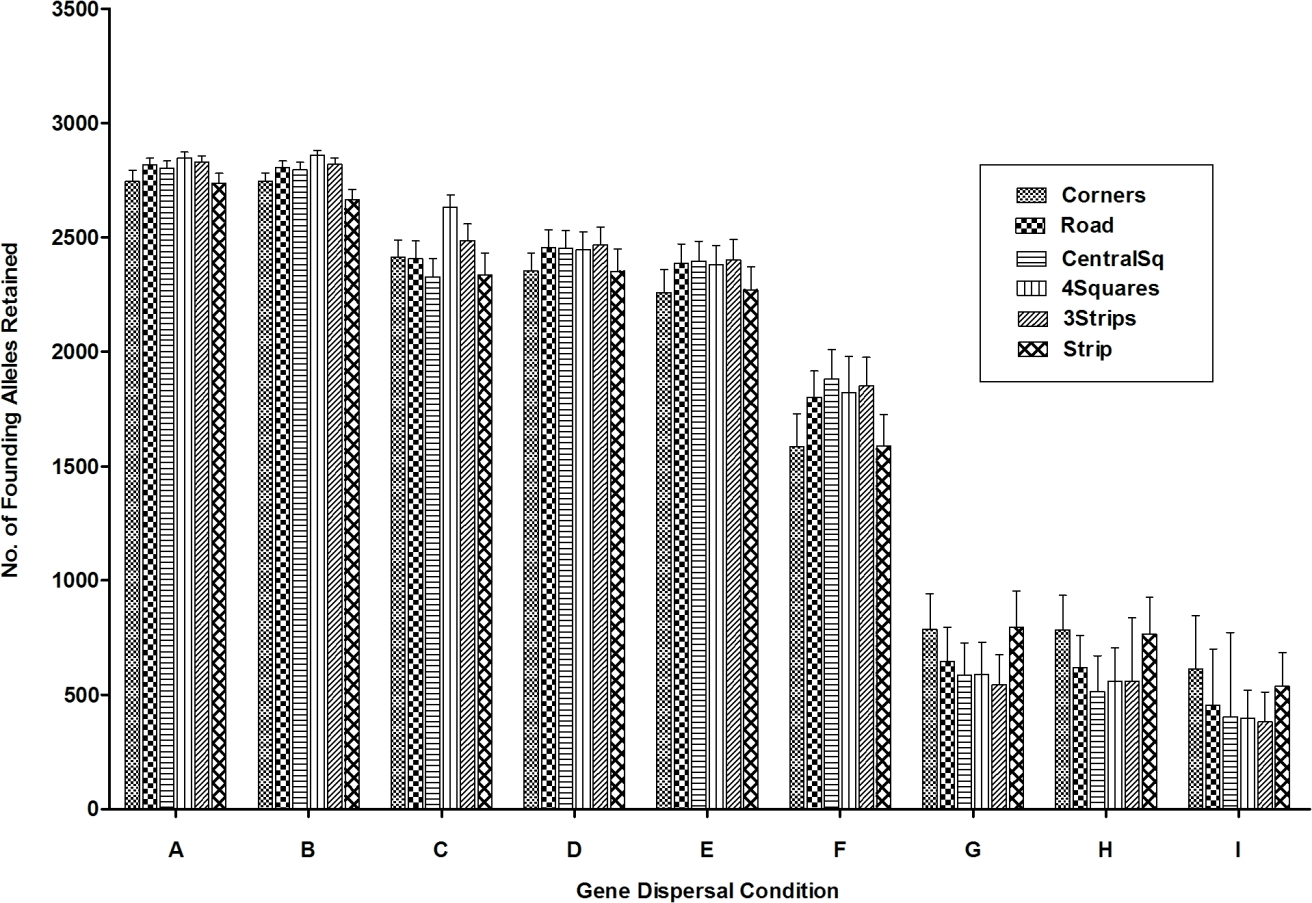
Mean founding allele retention after 600 generations for recovery regions (all logged areas combined). Trials varied in spatial logging patterns and gene dispersal conditions (offspring and pollen). Groups labelled A-I indicate trials grouped according to gene dispersal condition in descending order of mean population size: Gene dispersal condition is abbreviated as follows: ‘N’ = Near, ‘E’ = Equal, ‘F’ = Far, ‘P’ = Pollen, ‘O’ = Offspring, ‘Equal’, ‘Near’ and ‘Far’ refer to the probability of offspring and/or pollen being dispersed to or being received from a particular distance frame relative to a pistillate individual (for more detail see S1 Fig and S4 Table): A = NOEP; B = NONP; C = NOFP; D = EONP; E = EOEP; F = EOFP; G = FONP; H = FOEP; I = FOFP. “Control” refers to un-logged fragment, and “Selective Logging” refers to 10% removal of only those individuals belonging to the highest age class.

Compared to entire fragment results, there was a similar, but more pronounced decreasing trend for founding allele retention in recovery regions from offspring ‘Near’ dispersal trials to when offspring were dispersed to greater (‘Far’) distances (Fig 5). Variance explained by offspring, pollen dispersal and their interaction (as demonstrated by a two-way ANOVA), was similar to entire fragment trends (see S1 Appendix). Gene dispersal condition impacted allele retention the most, as a two-way ANOVA indicated that gene dispersal condition explained 97.3% of the variance (p<0.0001, F = 34685.28) relative to spatial logging pattern alterations (<0.01%, p<0.0001, F = 20.17) and the interaction of the two (0.8%, p<0.0001, F = 57.34; Fig 5). For gene dispersal groups A, B, D, E, and F (when offspring dispersal was either ‘Near’ or ‘Equal’), a greater retention of founding alleles for spatial logging patterns situated closer to the border (i.e., *Corners* and *Strip*) was found relative to more centrally located logging areas (*CentralSq*, *4Squares*, *3Strips*; Fig 5).

### Population subdivision/inbreeding—Entire fragment

**Fig 6:**
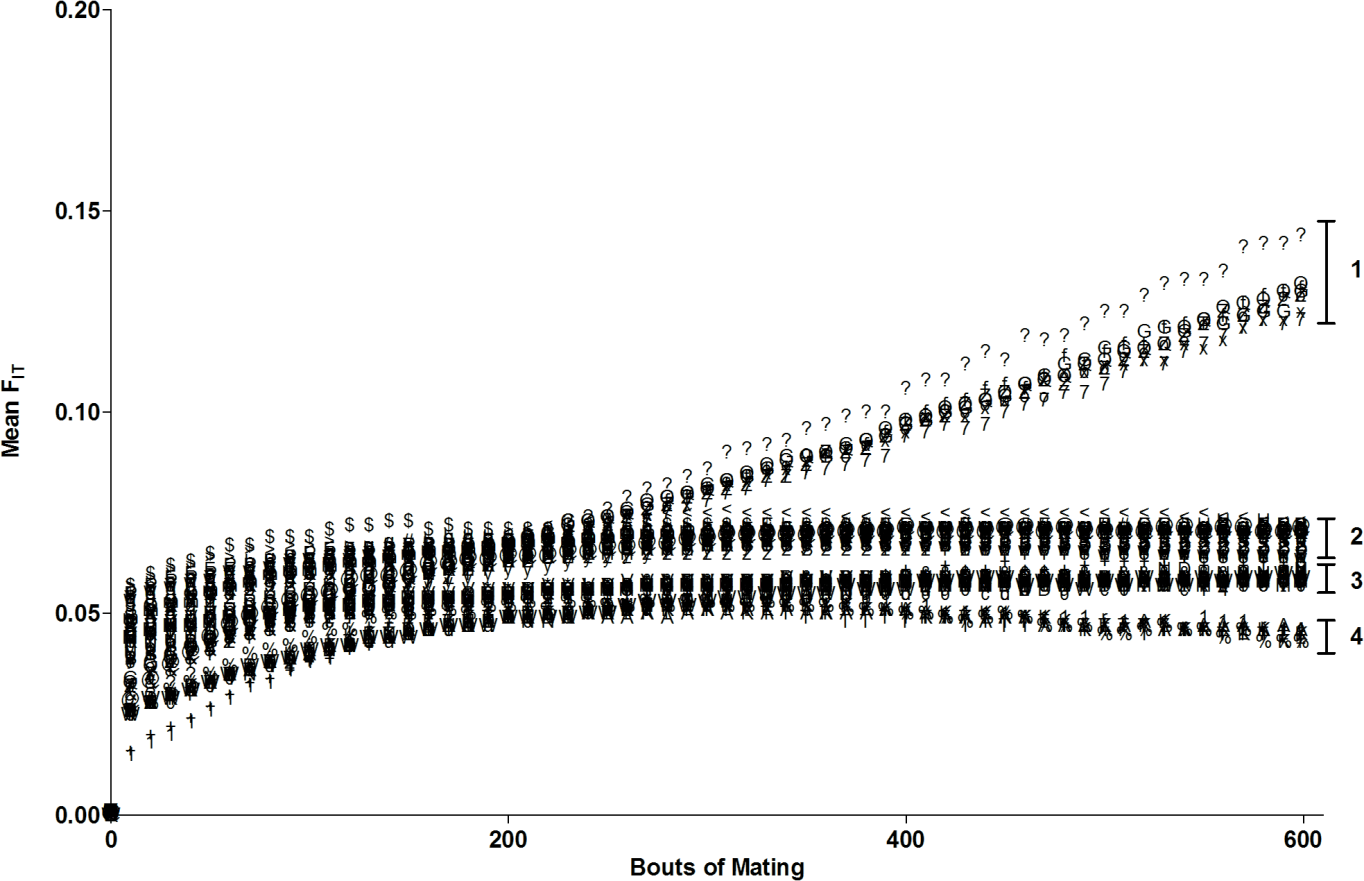
Mean Fit for trials across 600 bouts of mating in the entire fragment. Trials varied in spatial logging patterns and gene dispersal distance (offspring and pollen). Groups labelled 1–4 indicate trials grouped according to gene dispersal condition, with similar growth trajectories and endpoints. In group descending order of mean Fit levels: Gene dispersal condition is abbreviated as follows: ‘N’ = Near, ‘E’ = Equal, ‘F’ = Far, ‘P’ = Pollen, ‘O’ = Offspring, ‘Equal’, ‘Near’ and ‘Far’ refer to the probability of offspring and/or pollen being dispersed to or being received from a particular distance frame relative to a pistillate individual (for more detail S1 Fig and S4 Table); 1 = FONP; 2 = EONP; EOFP; NOFP; NONP; 3 = FOEP, EOEP, NOEP; 4 = FOFP. For a complete list of logging pattern and gene dispersal condition for each trial above, see S1 Appendix.

After 600 bouts of mating, F_it_ for the entire fragment for most dispersal condition groups and deforestation patterns indicated that moderate inbreeding/subdivision had occurred to varying degrees (Fig 6. and S4 Fig). Trials with ‘Near’ offspring and ‘Far’ pollen dispersal distances, (Group 1; Fig 6), showed increasing inbreeding and population subdivision, reaching moderate but increasing levels by the mating bout 600, with little sign that this trend would change with further bouts of mating. All other remaining gene dispersal conditions except for ‘Far’ offspring and pollen dispersal achieve a stable level of moderate inbreeding by approximately mating bout 175 (Groups 2 and 3; Fig 6). After 600 bouts of mating, trials varying in spatial logging patterns, and control trials (trials where no logging occurred) did not exhibit biologically meaningful differences in F_it_ values within gene dispersal groups (S4 Fig).

A two-way ANOVA analysis to demonstrate the relative effects of gene dispersal condition and spatial logging patterns on F_it_ levels showed that gene dispersal condition had the greatest impact (explaining 82.0% of the variance, p<0.0001, F = 4240.7, df = 8, N = 9) followed by the interaction of the two (0.6%, p<0.0001, F = 4.34, df = 56) and logging pattern respectively (0.2%, p<0.0001, F = 13.45, df = 7, N = 8; S4 Fig). A separate two-way ANOVA comparing the relative contributions of offspring versus pollen dispersal in explaining variation in F_it_ levels showed that the interaction of offspring and pollen dispersal had the greatest impact, explaining the highest mean variance (47.9%), followed by pollen dispersal (28.7%) and offspring dispersal (6.3%).

### Population subdivision/inbreeding—Recovery regions

F_it_ trajectories and endpoints were more complex in the recovery regions relative to the entire fragment (S5 Fig). Trials ended with essentially moderate levels of inbreeding/subdivision to varying degrees, except some trials with ‘Far’ offspring and ‘Near’ pollen (Group 1; S5 Fig) exhibited trajectories suggestive of greater F_it_ levels being reached in future generations. Although a two-way ANOVA showed that gene dispersal condition explained the most variance in F_it_ (38.0%, p<0.0001, F = 475.4, df = 8, N = 9) compared to the interaction (6.2%, p<0.0001, F = 15.55, df = 40), and logging pattern (2.4%, p<0.0001, F = 47.6, df = 5; S6 Fig), the magnitude of this variance was much less than in the entire fragment. One particular trial (*CentralSq*, ‘Near’ offspring and ‘Far’ pollen, trial z; S5 Fig) showed unusually high F_it_ values across early generations (ranging from 0.126–0.138) relative to all other trials. A separate two-way ANOVA analysis for demonstrating the relative effect of offspring versus pollen dispersal distance, showed that offspring dispersal distance had the greatest impact on F_it_ levels in the recovery regions, explaining a mean variance of 21.3%, followed by the interaction of the two dispersal conditions (14.7%) and pollen dispersal (10.1%).

## Discussion

This study explored the effects of variable spatial logging pattern and gene dispersal distances (via both offspring and pollen) on recovery population growth and genetic diversity dynamics for the plant functional type of a long-lived TLRF canopy tree species. Alternative combinations of logging pattern and gene dispersal distance affected this tropical population in different ways and to varying extents as described below. Different species and/or populations may respond quite distinctly to various extraction patterns depending on post-fragmentation dispersal attributes.

### Gene Dispersal condition

These simulations demonstrated how populations or similar species with different gene dispersal characteristics will respond differently to alternate disturbance patterns within fragments. Most of the variation in mean population size, retention of founding alleles, and population subdivision/inbreeding levels for both the entire fragments as well as recovery regions was attributable to differences in gene dispersal condition. In fragments of the size used here (500 ha) and conditions simulated in this study, populations exhibiting ‘Near’ offspring and pollen dispersal exhibited the highest population growth rates leading to greater levels of founding allele retention. Higher levels of rare allele retention have been found to be associated with lower variance in the frequencies of common alleles, making populations more tractable to the process of natural selection and less so to random genetic drift ([45], work in progress). Thus, changes in offspring and/or pollen dispersal distances brought about by fragmentation acting on genetic diversity retention, can potentially have consequences for the evolutionary trajectory of populations.

To the best of our knowledge, this is the first study to compare the effects of varying offspring versus pollen dispersal distances in TLRF fragments for this particular plant functional type. Our results indicate that offspring dispersal distance seems to be of greater importance relative to pollen dispersal and the interaction of the two dispersal types, in terms of its impact on population growth and founding allele retention as it explains a higher level of output variation. Nevertheless, variation in pollen dispersal distance can cause reduced but significant alterations in population growth and genetic diversity retention. The relative role of offspring dispersal is reduced for variation seen in population subdivision and/or inbreeding where the interaction of the two dispersal types takes on greater importance in the entire fragment. While pollen disperses genes in the haploid state, offspring genes are disseminated in the diploid state and thus contribute more to the genetic neighborhood size [68, 69].

The comparative trials here show that population differentiation and inbreeding for the entire fragment and recovery regions have a marked increase in F_it_ (to moderate levels) for the ‘Far’ offspring /‘Near’ pollen dispersal condition compared to all other trials, which exhibited little or no genetic spatial differentiation or inbreeding. When pollen is received primarily from ‘Near’ distances, there is a greater chances of locating eligible pollen donors for mating since the ‘Near’ dispersal distance range would fall within the fragment area and not outside of it. However, once mating occurs and offspring are produced, a relatively large proportion of these are dispersed to ‘Far’ distances off the fragment, leaving behind a small number of surviving individuals. The repetition of this process over time apparently leads to the creation of increasingly smaller and isolated subpopulations, which are more susceptible to increased inbreeding levels [70, 23]. The reverse of this gene dispersal condition (‘Near’ offspring and ‘Far’ pollen dispersal) does not, in this study, lead to such an increase in F_it_ values, suggesting the latter conditions are more effective in facilitating gene flow. These results demonstrate how varying offspring and pollen dispersal distances can interact to produce differences in the spatial genetic structure of a population [14].

Taken together, the results here suggest that changes in the abundance and/or behavior of pollinators and/or offspring dispersers occurring after fragmentation of populations of tropical long-lived canopy tree species, or the occurrence of such differences among species, will lead to complex and sometimes pronounced variation in the demographic trends and genetics for such populations or species going forward. NG-type analyses can be useful in predicting those species most at risk, and “best practices” for deforestation pattern, in fragments of a given size, although these simulations stress the importance of obtaining accurate life-history information (e.g., gene dispersal kernel estimates) for target species.

### Spatial logging pattern

Variations in spatial logging pattern explained lower levels of variance in population growth and genetic diversity levels compared to that accounted for by gene dispersal condition. This suggests that alterations in the spatial pattern of logging will not bring about large differences in resulting population recovery and population genetic dynamics, as would shifts in gene dispersal distances under the conditions modeled in this study. The most notable effect of spatial logging pattern detected from the results was that logging pattern frequently seemed to be of most consequence for population growth and founding allele retention in recovery regions when logging was situated closer to the border (e.g., *Corners*, *Strip*). Specifically, in five of the nine gene dispersal scenarios modeled, population recovery and allele retention were lower for deforestation occurring nearer borders.

Selective logging of the top 10% of the oldest or largest individuals tended to yield the lowest rates of population recovery and founding allele retention relative to the other extraction patterns. This supports the notion that moderate harvesting of non-timber products obtainable from a range of size-class groups could be more sustainable than harvesting for the largest trees only, as the latter puts greater pressure on the more reproductive size-classes [71].

### Conservation implications

NG modeling can be used to model population trends under different fragment sizes, gene dispersal conditions, deforestation patterns, or other life-history characteristics that can be characterized in NG input, for species of concern. Since trends in population growth, genetic diversity retention, and/or inbreeding and subdivision here were, in some cases, highly sensitive to post-fragmentation alterations in gene dispersal conditions, one practical management technique might include interventions to potentially alter offspring and/or pollen dispersal distances (manual manipulations or alteration of dispersal vector agents) to reflect those that maximize population size and the retention of genetic diversity for the population and conditions simulated in this study. Even if a newly established fragment has not undergone internal deforestation, any subsequent changes in gene dispersal distances due to fragmentation can affect population sizes and genetic diversity retention to a great extent as suggested by the results from the control trials simulating unlogged fragments under varying gene dispersal conditions.

In this study, which explores the effects of both spatial logging pattern and gene dispersal distances (including both offspring and pollen dispersal distances), it was simpler to begin with a control population initially in genetic equilibrium in a spatially uniform landscape to determine the general effects of these variables separately, as well their synergistic effects. We refrained from choosing one of the numerous alternative population-genetic and/or spatially heterogeneous scenarios that could be modeled, as this would have been an arbitrary decision and made interpretation more complex. Nevertheless, a multitude of other complex non-equilibrium population and genetic history scenarios that may have occurred at different locations (e.g., genetic bottlenecks, founder effects etc.) as well as different kinds of spatially heterogeneous landscapes can be modeled by NEWGARDEN when site-specific information is available concerning the various user-defined input parameters. For example, if the history of a particular forest fragment is known or suspected, NEWGARDEN can be used to modify the characteristics of the initial population and geographical aspects of the forest to explore the demographic and population genetic consequences of such modifications. As tropical low land rain forests share similar general abiotic (e.g., precipitation and solar radiation levels, humidity, etc.) and biotic (low population densities, species diversity in guilds of offspring and pollen dispersers servicing plant taxa, soil microbes, etc.) characteristics, the general scenarios and conclusions described from this study for this plant functional type are not confined to one region of the tropics, but theoretically to tropical low land rain forests across the equatorial belt [72]. The diversity of the life-history characteristics of TLRF trees suggests that fragmented and deforested populations may exhibit a wide variety of intra- and interspecific responses. Thus, we emphasize that the trial comparisons here are not meant to be exhaustive. Rather, population responses under scenarios where numerous other features vary, such as fragment sizes, shapes, establishment densities, gene dispersal or other life history characteristics, removal regimens, etc., can be explored with NG.

## Supporting Information

S1 Appendix This file contains detailed methods which further elaborate on how the NEWGARDEN program functions and the life-history characteristics of the simulated population. It also contains supporting information from the results section as well the complete figure legends for Figs 2 and 6, and the supporting figures S2 Fig and S5 Fig
(DOCX)

S1 Dataset This file contains the raw datasets from this research study. (XLSX)

S1 Fig Nested offspring and pollen dispersal distance frames around a hypothetical offspring-producing individual (“x”) selected for mating. This individual can be situated anywhere in the 500 ha fragment. The following distances are relative to individual “x” in the x and y direction: a = 32 grid points or 448 m, b = 64 grid points or 896 m, c = 96grid points or 1344 m, d = 128 grid points or 1792 m, e = 160 grid points or 2240 m. Diagram is approximately to scale.
(TIF)

S2 Fig Mean population growth across 600 generations in recovery regions (all logged areas combined). Trials varied in spatial logging patterns and gene dispersal distance (offspring and pollen). Groups labelled A-I indicate trials grouped according to gene dispersal condition, with similar growth trajectories and endpoints. In descending order of mean population size: Logging pattern description is followed by gene dispersal condition abbreviated as follows: ‘N’ = Near, ‘E’ = Equal, ‘F’ = Far, ‘P’ = Pollen, ‘O’ = Offspring, ‘Equal’, ‘Near’ and ‘Far’ refer to the probability of offspring and/or pollen being dispersed to or being received from a particular distance frame relative to a pistillate individual (for more detail see S1 Fig and S4 Table); A = NONP, B = NOEP, C = NOFP, D = EONP, E = EOEP, F = EOFP, G = FONP, FOEP, FOFP. For a complete list of logging pattern and gene dispersal condition for each trial above, see S1 Appendix.
(TIF)

S3 Fig Mean founding allele retention after 600 bouts of mating in the entire fragment. Trials varied in spatial logging patterns and gene dispersal distance (offspring and pollen). Groups labelled A-I indicate trials grouped according to gene dispersal condition in descending order of mean population size: Logging pattern description is followed by gene dispersal condition abbreviated as follows: ‘N’ = Near, ‘E’ = Equal, ‘F’ = Far, ‘P’ = Pollen, ‘O’ = Offspring, ‘Equal’, ‘Near’ and ‘Far’ refer to the probability of offspring and/or pollen being dispersed to or being received from a particular distance frame relative to a pistillate individual (for more detail see S1 Fig and S4 Table); A = NONP, B = NOEP, C = NOFP, D = EONP, E = EOEP, F = EOFP, G = FONP, H = FOEP, I = FOFP. “Control” refers to un-logged fragment, and “Selective Logging” refers to 10% removal of only those individuals belonging to the highest age classes.
(TIF)

S4 Fig Mean F_it_ after 600 bouts of mating in the entire fragment. Trials varied in spatial logging patterns and gene dispersal conditions (offspring and pollen). Groups labelled 1–9 indicate trials grouped according to descending order of mean population, with spatial logging patterns in the legends appearing from left to right within each gene dispersal scenario: Gene dispersal conditions are abbreviated as follows: ‘N’ = Near, ‘E’ = Equal, ‘F’ = Far, ‘P’ = Pollen, ‘O’ = Offspring, ‘Equal’, ‘Near’ and ‘Far’ refer to the probability of offspring and/or pollen being dispersed to or being received from a particular distance frame relative to a pistillate individual (for more detail see S1 Fig and S4 Table); 1 = FONP, 2 = EOFP, 3 = EONP, 4 = NOFP, 5 = NONP, 6 = FOEP, 7 = EOEP, 8 = NOEP, 9 = FOFP. “Control” refers to un-logged fragment, and “Selective Logging” refers to 10% removal of only those individuals belonging to the highest age classes.
(TIF)

S5 Fig Mean F_it_ across 600 bouts of mating for recovery regions (all logged areas combined). Trials varied in spatial logging patterns and gene dispersal distance (offspring and pollen dispersal). Group 1 indicates trials grouped according to gene dispersal condition, with similar growth trajectories and endpoints. In descending order of mean population size: Logging pattern description is followed by gene dispersal condition abbreviated as follows: ‘N’ = Near, ‘E’ = Equal, ‘F’ = Far, ‘P’ = Pollen, ‘O’ = Offspring, ‘Equal’, ‘Near’ and ‘Far’ refer to the probability of offspring and/or pollen being dispersed to or being received from a particular distance frame relative to a pistillate individual (for more detail see S1 Fig and S4 Table); 1 = FONP. For a complete list of logging pattern and gene dispersal condition for each trial above, see S1 Appendix.
(TIF)

S6 Fig Mean F_it_ after 600 bouts of mating for recovery regions (all logged areas combined). Trials varied in spatial logging patterns and gene dispersal distance (offspring and pollen). Groups labelled 1–9 indicate trials grouped according to gene dispersal condition in descending order of mean population: Gene dispersal conditions are abbreviated as follows, with spatial logging pattern in the legend appearing from left to right within each gene dispersal scenario: ‘N’ = Near, ‘E’ = Equal, ‘F’ = Far, ‘P’ = Pollen, ‘O’ = Offspring, ‘Equal’, ‘Near’ and ‘Far’ refer to the probability of offspring and/or pollen being dispersed to or being received from a particular distance frame relative to a pistillate individual (S1 Fig and S4 Table); 1 = FONP, 2 = FOFP, 3 = FOEP, 4 = NOFP, 5 = EOFP, 6 = EONP, 7 = EOEP, 8 = NONP, 9 = NOEP. “Control” refers to un-logged fragment, and “Selective Logging” refers to 10% removal of only those individuals belonging to the highest age classes.
(TIF)

S1 Table Similarities and differences between NEWGARDEN (NG) and other forest growth models including stand models, process-based models, empirical models, gap models and hybrid models. (DOCX)

S2 Table Age-specific rates of reproduction and pollen provisioning for the control equilibrium population. (DOCX)

S3 Table Age-specific mortality rate for the control equilibrium population. (DOCX)

S4 Table Offspring and pollen dispersal distance dimensions. Distance frame dimensions and selection probabilities for offspring and pollen dispersal distance from or to (respectively) an offspring-producing individual in a given mating event for the “Equal”, “Near” and “Far” gene dispersal categories.
(DOCX)
