# Supplementary material for: Variable Gene Dispersal Conditions and Spatial Deforestation Patterns Can Interact to Affect Tropical Tree Conservation Outcomes
Source: PLoS One. 2015 May 22;10(5):e0127745. doi: 10.1371/journal.pone.0127745 (PMC4441416; doi:10.1371/journal.pone.0127745)
Supplement: S1 Appendix — It also contains supporting information from the results section as well the complete figure legends for Figs 2 and 6, and the supporting figures S2 Fig and S5 Fig (DOCX) [file pone.0127745.s001.docx]

**S1 Appendix**

This file contains detailed methods which further elaborate on how the NEWGARDEN program functions and the life-history characteristics of the simulated population. It also contains supporting information from the results section as well the complete figure legends from figures 2 and 6, and the supporting figures S2 Fig. and S5 Fig.

**Methods**

**Control Equilibrium Population**

**Age-class distribution**

Size-class distribution of the initial founding population was based on field data on *S. globulifera* obtained from a study by Carneiro et al. 2009 [1]. This distribution was converted to corresponding age classes based on average growth rates reported from studies at Pasoh Forest Reserve, Malaysia, with growth rates ranging from 2 to 4 mm per year for seedlings and juveniles, and roughly 4 to 7 mm/year for mature ages [2], averaged across numerous TLRF tree species. During simulations, NG would randomly assign founders ages according to this derived age-class distribution. As only those plants with a DBH of greater than 15cm were censused in [1] to model the younger size classes, size class distribution data from a canopy species (*Cynometra malaccensis* Meeuwen (Fabaceae)) in Pasoh Forest Reserve, Malaysia, was used to create a representative distribution [3]. Tropical climax species have been reported to have size class distributions which have fewer and fewer individuals in each successive size class [3, 4]. Thus, lower size classes make up the largest portion of the population and thereby significantly impact resulting demographic and genetic establishment patterns [5].

**Mortality**

In NG, users can specify age-specific rate of mortality for the developing population. In addition to this method of ‘dying’, individuals may “die”, meaning they are removed from subsequent censuses used to generate output statistics if: they are dispersed off the fragment; two individuals land on the same grid point, after which one of them is randomly selected to establish while the other is removed; an individual is randomly selected to be removed according to the specified mortality rate; an individual tries to establish on an occupied grid point; or if an ovule fails to be pollinated due to lack of an eligible pollen donor [6].

We used age-specific mortality curves representing long-lived canopy tree species described by Metcalf et al. 2009 in La Selva, Costa Rica [7]. In summary, representative patterns followed a “bath-tub” shaped curve, with much higher mortality rates at younger ages [8], and mortality diminishing to the lowest levels at the “juvenile” stage. Thereafter, mortality rates increase slightly and plateau at intermediate ages, and finally decline at the onset of senescence [7].

**Results**

**Founding allele retention – Entire fragment**

Founding allele retention in the entire fragment and the recovery region was maximized for the ‘Near’ offspring dispersal conditions (Groups A-C; S3 Fig. and Fig. 5), followed by ‘Equal’ dispersal (Groups D-F; S3 Fig. and Fig. 5) and ‘Near’ (Groups G-I; S3 Fig. and Fig. 5). For the entire fragment and recovery regions, when offspring dispersal distance was constant, groups were arranged starting with ‘Near’ pollen, followed by ‘Equal’ and ‘Far’ pollen respectively (S3 Fig. and Fig. 5).

**Founding allele retention – Recovery region**

A two-way ANOVA showed that offspring dispersal explained a greater amount of variance in mean founding allele retention for the recovery regions (mean variance of 92.9%), relative to pollen dispersal (4.2%) and the interaction (1.0%).

**Mean observed heterozygosity – Entire fragment**

(As this output was not as sensitive to variations in gene dispersal condition and logging pattern as was founding allele retention, we used the latter as a measure for genetic diversity. Following are general details for this output).

Variations in gene dispersal condition and logging pattern yielded more or less ‘high’ mean H_o_ values, ranging from 0.831 to 0.931 in the entire fragment. Trials with ‘Near’ offspring and ‘Far’ pollen dispersal had the lowest mean H_o_ compared to all other gene dispersal groups. Throughout the 600 generations, this particular gene dispersal condition follows a markedly decreasing H_o_ trajectory, with an increasing trend for F_it_, indicating possible effects of moderate levels of population subdivision for this gene dispersal group.

**Mean observed heterozygosity – Recovery Region**

In the combined logged areas, mean H_o_ levels ranged from 0.819 to 0.926. ‘Near’ offspring and ‘Far’ pollen dispersed trials showed a declining H_o_ trajectory and endpoints compared to all other trials, similar to trends seen in the entire fragment. This gene dispersal group also had increasing F_it_ levels reaching moderate population differentiation.

**Figure legends in full**

**Fig. 2** In descending order of mean population size after 600 bouts of mating in the entire fragment: Logging pattern description is followed by gene dispersal condition abbreviated as follows: ‘N’= Near, ‘E’= Equal, ‘F’=Far, ‘P’=Pollen, ‘O’= Offspring , ‘Equal’, ‘Near’ and ‘Far’ refer to the probability of offspring and/or pollen being dispersed to or being received from a particular distance frame relative to a pistillate individual (for more detail see S3 Table and S1 Fig.). **Group A= NONP** (6= Control NONP, Y= Strip NONP, e= Corner NONP, w= CentralSq NONP, n= Road NONP, F= 4Squares NONP, P= 3Strips NONP), 3= Control NOEP; **Group B= NOEP** (b= Corner NOEP), >= Selective Logging NONP; Group B**=** NOEP (k= Road NOEP, M= 3Strips NOEP, V= Strip NOEP, t= CentralSq NOEP, C= 4Squares NOEP), *= Selective Logging NOEP; **Group C= NOFP** (9= Control NOFP, z= CentralSq NOFP, S= 3Strips NOFP, J= 4Squares NOFP, h= Corner NOFP, q= Road NOFP, $= Strip NOFP, != Selective Logging NOFP); **Group D= EONP** (5= Control EONP, X= Strip EONP, O= 3Strips EONP, d= Corner EONP, m= Road EONP, E= 4Squares EONP, v= CentralSq EONP) , 2= Control EOEP, <= Selective Logging EONP; **Group E= EOEP** (L= 3Strips EOEP, U= Strip EOEP, a= Corner EOEP, s= CentralSq EOEP, j= Road EOEP, B= 4Squares EOEP, &= Selective Logging EOEP); **Group F= EOFP** (8= Control EOFP, y= CentralSq EOFP, R= 3Strips EOFP, p= Road EOFP, H= 4Squares EOFP, g= Corner EOFP, #= Strip EOFP, @= Selective Logging EOFP); **Group G,H,I = FONP, FOEP, FOFP** (A= CentralSq FOFP, N= 3Strips FOEP, x= CentralSq FONP,7= Control FONP, G= 4Squares FONP, o= Road FONP, 4= Control FOEP, Q= 3Strips FONP, Z= Strip FONP, u= CentralSq FOEP, l= Road FOEP, D= 4Squares FOEP, r= Road FOFP, f= Corner FONP, W= Strip FOEP, c= Corner FOEP, ?= Selective Logging FONP, = = Control FOFP, += Selective Logging FOEP, T= 3Strips FOFP, K= 4Squares FOFP, i= Corner FOFP, %= Strip FOFP, 1= Selective Logging FOFP).

**Fig. 6** In descending order of mean F_it_ levels after 600 bouts of mating in the entire fragment: Logging pattern description is followed by gene dispersal condition abbreviated as follows: ‘N’= Near, ‘E’= Equal, ‘F’=Far, ‘P’=Pollen, ‘O’= Offspring , ‘Equal’, ‘Near’ and ‘Far’ refer to the probability of offspring and/or pollen being dispersed to or being received from a particular distance frame relative to a pistillate individual (for more detail see S3 Table and S1 Fig.); **Group 1= FONP** (?= Selective Logging FONP, 7= Control FONP, Q= 3Strips FONP, f= Corner FONP,O= Road FONP, Z= Strip FONP, G= 4Squares FONP, x= CentralSq FONP); **Group 2= EONP, EOFP, NOFP, NONP** (<= Selective Logging EONP, R= 3Strips EOFP, H= 4Squares EOFP, g= Corner EOFP, @= Selective Logging EOFP, #= Strip EOFP, E= 4Squares EONP, v= CentralSq EONP, m= Road EONP, 8= Control EOFP, $= Strip NOFP, X= Strip EONP, O= 3Strips EONP, y= CentralSq EOFP, p= Road EOFP, h= Corner NOFP, d= Corner EONP, 9= Control NOFP, q Road NOFP, 5= Control EONP, != Selective Logging NOFP, S= 3Strips NOFP, J= 4Squares NOFP, >= Selective Logging NONP, z= CentralSq NOFP, P= 3Strips NONP,F= 4Squares NONP, w= CentralSq NONP, 6= Control NONP, Y= Strip NONP, n= Road NONP, e= Corner NONP); **Group 3= FOEP, EOEP, NOEP** (+= Selective Logging FOEP, u= CentralSq FOEP, 4= Control FOEP, 2= Control EOEP, &= Selective Logging EOEP, W= Strip FOEP, N= 3Strips FOEP, U= Strip EOEP, L= 3Strips EOEP, D= 4Squares EOEP, B= 4Squares EOEP, s= CentralSq EOEP, l= Road FOEP, a= Corner EOEP, 3= Control NOEP, *= Selective Logging NOEP, V= Strip NOEP, C= 4Squares NOEP, k= Road NOEP, j= Road EOEP, c= Corner FOEP, b= Corner NOEP, M= 3Strips NOEP, t= CentralSq NOEP; **Group 4=FOFP** (A= CentralSq FOFP, 1= Selective Logging FOFP, r= Road FOFP, i= Corner FOFP, = = Control FOFP, T= 3Strips FOFP, %= Strip FOFP, K= 4Squares FOFP).

**S2 Fig. Group A = NONP** (F= 4Squares NONP, P= 3Strips NONP, w= CentralSq NONP, n= Road NONP); **Group B= NOEP** (C= 4Squares NOEP, M= 3Strips NOEP, k= Road NOEP, t= CentralSq NOEP), Y= Strip NONP, J= 4Squares NOFP, V= Strip NOEP, e= Corner NONP, b= Corner NOEP; **Group C= NOFP** (h= Corner NOFP, q= Road NOFP, $= Strip NOFP, S= 3Strips NOFP); **Group D= EONP** (v= CentralSq EONP, O= 3Strips EONP, m= Road EONP, E= 4Squares EONP); **Group E= EOEP** (s= CentralSq EOEP, L= 3Strips EOEP, j= Road EOEP, B= 4Squares EOEP), X= Strip EONP, D= Corner EONP, z= CentralSq NOFP, U= Strip EOEP, a= Corner EOEP; **Group F= EOFP** (R= 3Strips EOFP, y= CentralSq EOFP, H= 4Squares EOFP, p= Road EOFP, #= Strip EOFP, g= Corner EOFP); **Group G= FONP, FOEP, FOFP** (f= Corner FONP, c= Corner FOEP, Z= Strip FONP, W= Strip FOEP, i= Corner FOFP, A= CentralSq FOFP, N= 3Strips FOEP, %= Strip FOFP, o= Road FONP, l= Road FOEP, x= CentralSq FONP, G= 4Squares FONP, Q= 3Strips FONP, r= Road FOFP, D= 4Squares FOEP, u= CentralSq FOEP, K= 4Squares FOFP, T= 3Strips FOFP

**S5 Fig. Group 1= FONP (**f= Corner FONP, Q= 3Strips FONP, Z= Strip FONP, G= 4Squares FONP, o= Road FONP, x= CentralSq FONP), z= CentralSq NOFP, A= CentralSq FOFP, T= 3Strips FOFP, K= 4Squares FOFP, N= 3Strips FOEP, D= 4Squares FOEP, u= CentralSq FOEP, y= CentralSq EOFP, r= Road FOFP, S= 3Strips NOFP, l= Road FOEP, R= 3Strips EOFP, p= Road EOFP, H= 4Squares EOFP, W= Strip FOEP, c= Corner FOEP, J= 4Squares NOFP, d= Corner EONP, X= Strip EONP, E= 4Squares EONP, m= Road EONP, O= 3Strips EONP, v= CentralSq EONP, e= Corner NONP, #= Strip EOFP, t= CentralSq NOEP, Y= Strip NONP, L= 3Strips EOEP, F= 4Squares NONP, g= Corner EOFP, P= 3Strips NONP, s= CentralSq EOEP, w= CentralSq NONP, n= Road NONP, j= Road EOEP, M= 3Strips NOEP, B= 4Squares EOEP, U= Strip EOEP, k= Road NOEP, a= Corner EOEP, C= 4Squares NOEP, i= Corner FOFP, q= Road NOFP, b= Corner NOEP, $= Strip NOFP, V= Strip NOEP, h= Corner NOFP, %= Strip FOFP

**References**

1. Carneiro FD, Degen B, Kanashiro M, de Lacerda AE, Sebbenn, AM (2009) High levels of pollen dispersal detected through paternity analysis from a continuous *Symphonia globulifera* population in the Brazilian Amazon. Forest Ecology and Management 258: 1260-1266.
2. Condit R, Ashton PS, Manokaran N, LaFrankie JV, Hubbell SP, et al. (1999) Dynamics of the forest communities at Pasoh and Barro Colorado: comparing two 50-ha plots. Phil. Trans. R. Soc. Lond. B 354: 1739-1748.
3. Forest Research Institute of Malaysia (FRIM) (1993) Distribution of Tree Species in the Fifty Hectare Research Plot at Pasoh Forest Reserve. Selangor Darul Ehsan: FRIM. 454pp.
4. Jennings S, Brown N, Boshier D, Whitmore T, Lopes J (2001) Ecology provides a pragmatic solution to the maintenance of genetic diversity in sustainably managed tropical rain forests. Forest Ecology and Management 154: 1-10.
5. Alvarez-Buyalla ER, Garcia-Barrios R, Lara-Moreno C, Martinez-Ramos M (1996). Demographic and genetic models in conservation biology: applications and perspectives for tropical rain forest tree species. Annual Review of Ecology and Systematics 27: 387-421.
6. Rogstad SH, Pelikan S (2011)  Genetic diversity in establishing plant populations: Founder number and geometry. Enfield: Science Publishers. 360 pp.
7. Metcalf C, Horvitz C, Tuljapurkar S, Clark D (2009) A time to grow and a time to die: a new way to analyze the dynamics of size, light, age, and death of tropical trees. Ecology 90: 2766-2778.
8. Augspurger C, Kitajima K (1992) Experimental studies of seedling recruitment from contrasting seed distributions. Ecology 73: 1270-1284.
