# Supplementary figures and images for: Variable Gene Dispersal Conditions and Spatial Deforestation Patterns Can Interact to Affect Tropical Tree Conservation Outcomes
Source: PLoS One. 2015 May 22;10(5):e0127745. doi: 10.1371/journal.pone.0127745 (PMC4441416; doi:10.1371/journal.pone.0127745)

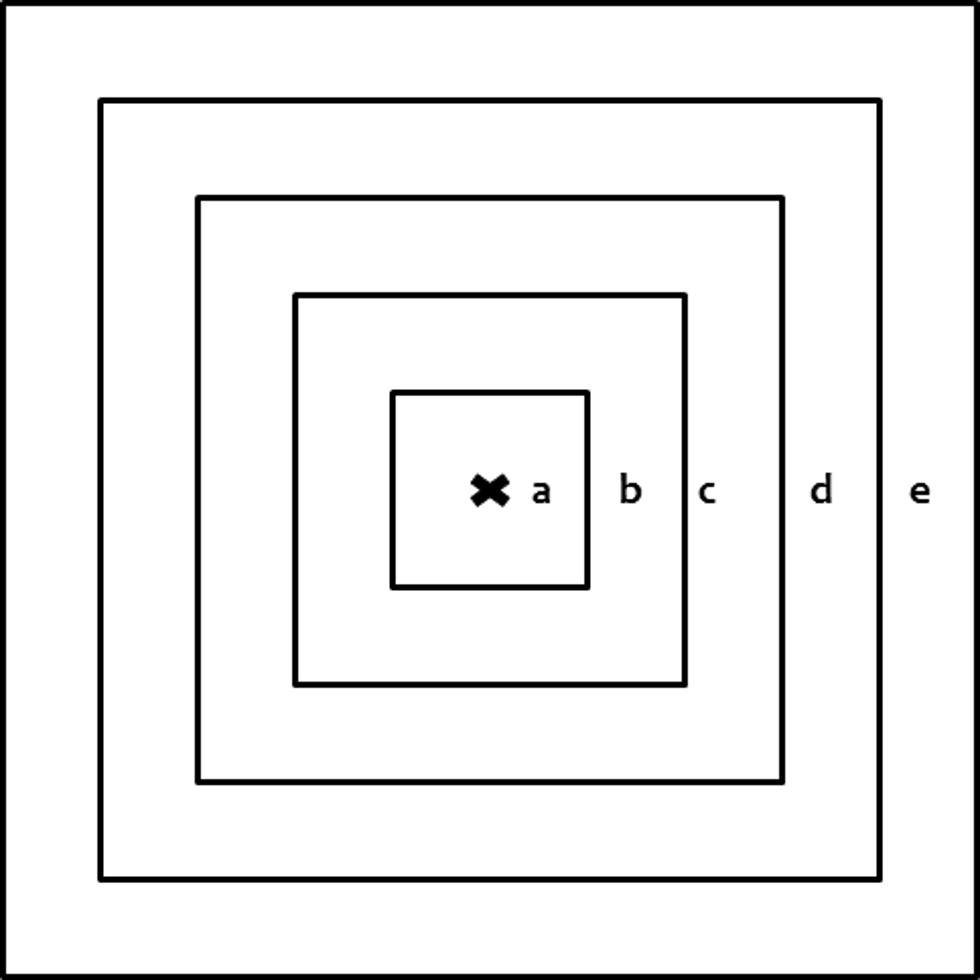

Supplement: S1 Fig — This individual can be situated anywhere in the 500 ha fragment. The following distances are relative to individual “x” in the x and y direction: a = 32 grid points or 448 m, b = 64 grid points or 896 m, c = 96grid points or 1344 m, d = 128 grid points or 1792 m, e = 160 grid points or 2240 m. Diagram is approximately to scale. (TIF) [file pone.0127745.s003.tif]

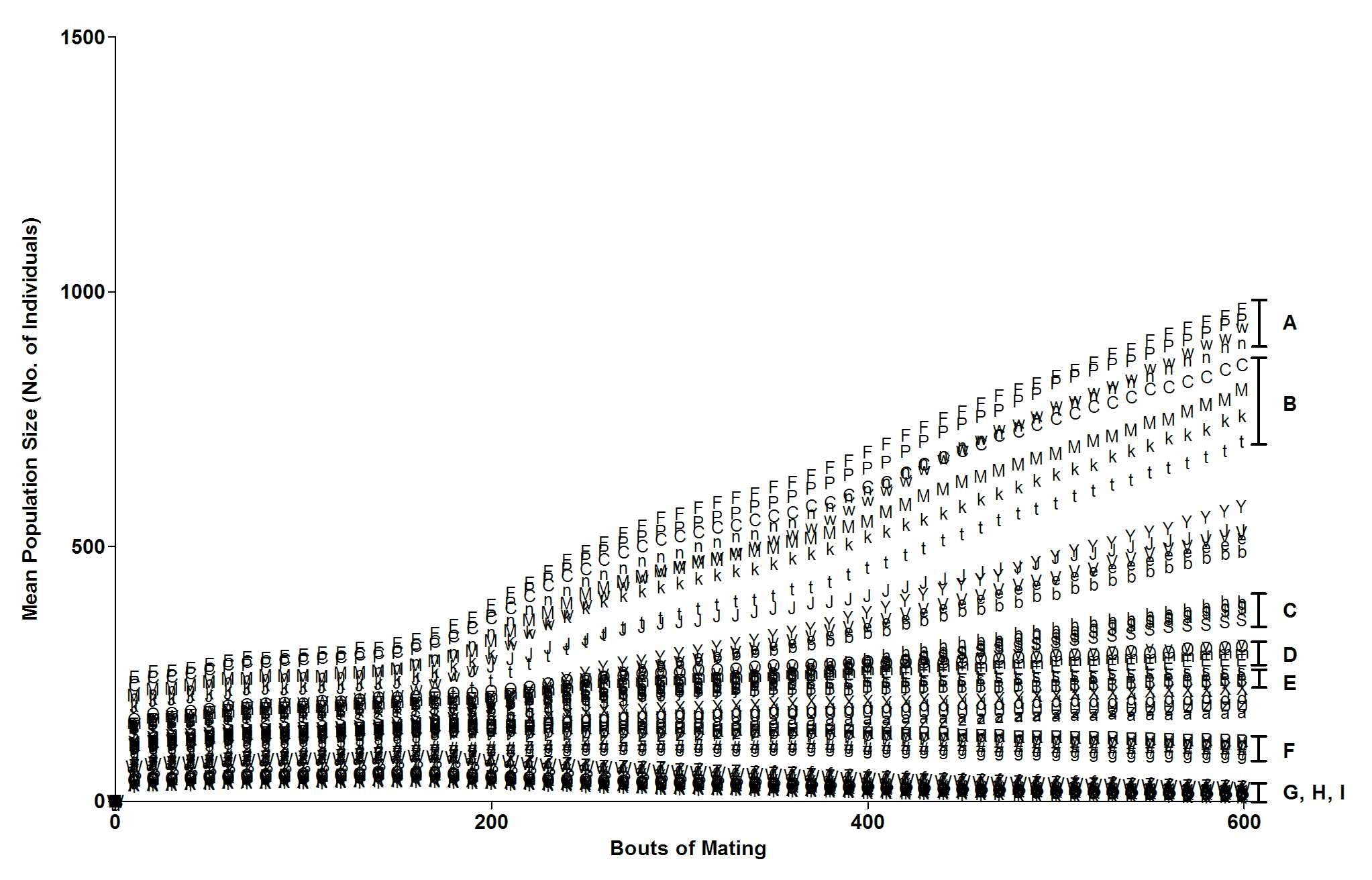

Supplement: S2 Fig — Trials varied in spatial logging patterns and gene dispersal distance (offspring and pollen). Groups labelled A-I indicate trials grouped according to gene dispersal condition, with similar growth trajectories and endpoints. In descending order of mean population size: Logging pattern description is followed by gene dispersal condition abbreviated as follows: ‘N’ = Near, ‘E’ = Equal, ‘F’ = Far, ‘P’ = Pollen, ‘O’ = Offspring, ‘Equal’, ‘Near’ and ‘Far’ refer to the probability of offspring and/or pollen being dispersed to or being received from a particular distance frame relative to a pistillate individual (for more detail see S1 Fig and S4 Table); A = NONP, B = NOEP, C = NOFP, D = EONP, E = EOEP, F = EOFP, G = FONP, FOEP, FOFP. For a complete list of logging pattern and gene dispersal condition for each trial above, see S1 Appendix. (TIF) [file pone.0127745.s004.tif]

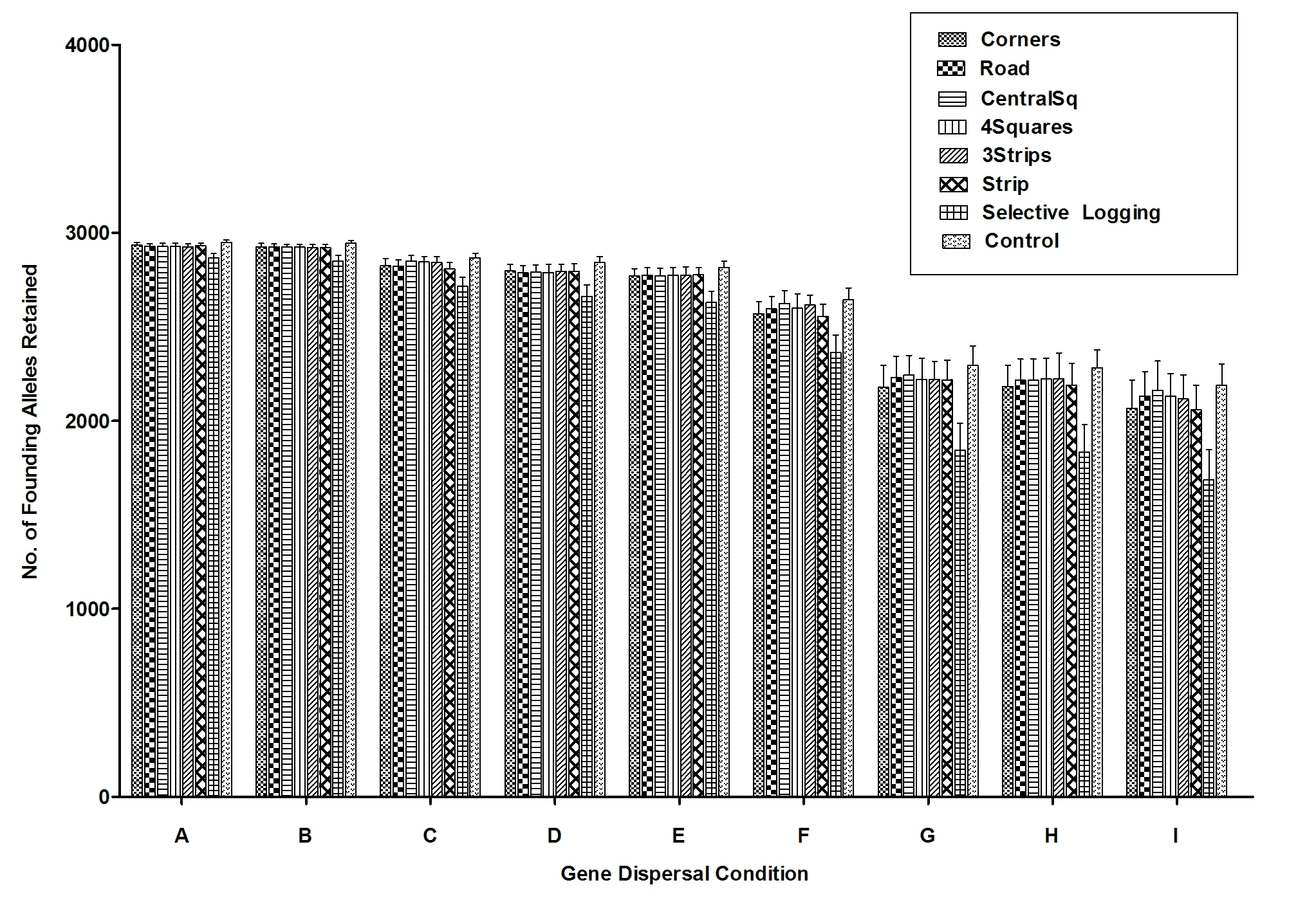

Supplement: S3 Fig — Trials varied in spatial logging patterns and gene dispersal distance (offspring and pollen). Groups labelled A-I indicate trials grouped according to gene dispersal condition in descending order of mean population size: Logging pattern description is followed by gene dispersal condition abbreviated as follows: ‘N’ = Near, ‘E’ = Equal, ‘F’ = Far, ‘P’ = Pollen, ‘O’ = Offspring, ‘Equal’, ‘Near’ and ‘Far’ refer to the probability of offspring and/or pollen being dispersed to or being received from a particular distance frame relative to a pistillate individual (for more detail see S1 Fig and S4 Table); A = NONP, B = NOEP, C = NOFP, D = EONP, E = EOEP, F = EOFP, G = FONP, H = FOEP, I = FOFP. “Control” refers to un-logged fragment, and “Selective Logging” refers to 10% removal of only those individuals belonging to the highest age classes. (TIF) [file pone.0127745.s005.tif]

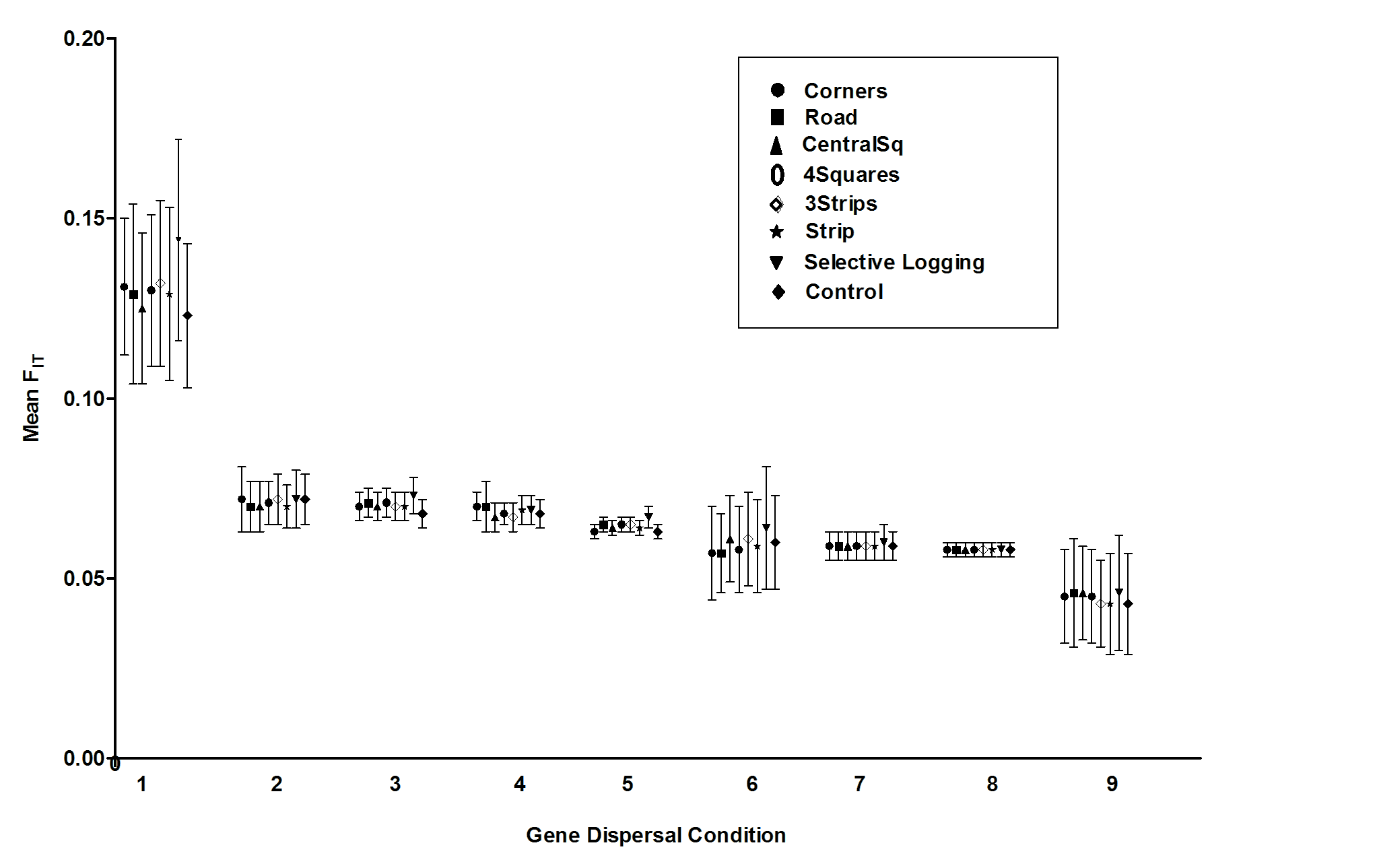

Supplement: S4 Fig — Trials varied in spatial logging patterns and gene dispersal conditions (offspring and pollen). Groups labelled 1–9 indicate trials grouped according to descending order of mean population, with spatial logging patterns in the legends appearing from left to right within each gene dispersal scenario: Gene dispersal conditions are abbreviated as follows: ‘N’ = Near, ‘E’ = Equal, ‘F’ = Far, ‘P’ = Pollen, ‘O’ = Offspring, ‘Equal’, ‘Near’ and ‘Far’ refer to the probability of offspring and/or pollen being dispersed to or being received from a particular distance frame relative to a pistillate individual (for more detail see S1 Fig and S4 Table); 1 = FONP, 2 = EOFP, 3 = EONP, 4 = NOFP, 5 = NONP, 6 = FOEP, 7 = EOEP, 8 = NOEP, 9 = FOFP. “Control” refers to un-logged fragment, and “Selective Logging” refers to 10% removal of only those individuals belonging to the highest age classes. (TIF) [file pone.0127745.s006.tif]

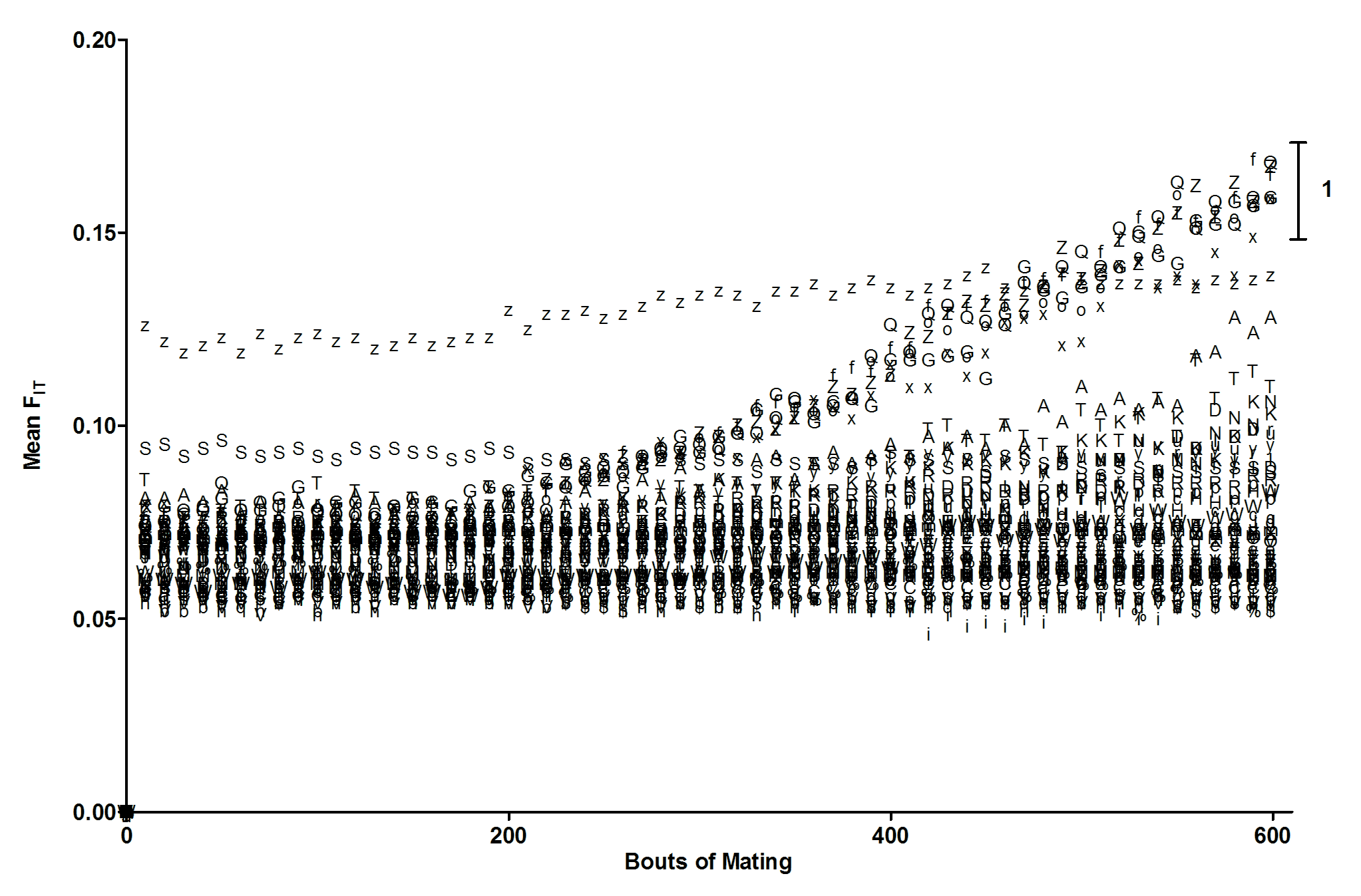

Supplement: S5 Fig — Trials varied in spatial logging patterns and gene dispersal distance (offspring and pollen dispersal). Group 1 indicates trials grouped according to gene dispersal condition, with similar growth trajectories and endpoints. In descending order of mean population size: Logging pattern description is followed by gene dispersal condition abbreviated as follows: ‘N’ = Near, ‘E’ = Equal, ‘F’ = Far, ‘P’ = Pollen, ‘O’ = Offspring, ‘Equal’, ‘Near’ and ‘Far’ refer to the probability of offspring and/or pollen being dispersed to or being received from a particular distance frame relative to a pistillate individual (for more detail see S1 Fig and S4 Table); 1 = FONP. For a complete list of logging pattern and gene dispersal condition for each trial above, see S1 Appendix. (TIF) [file pone.0127745.s007.tif]

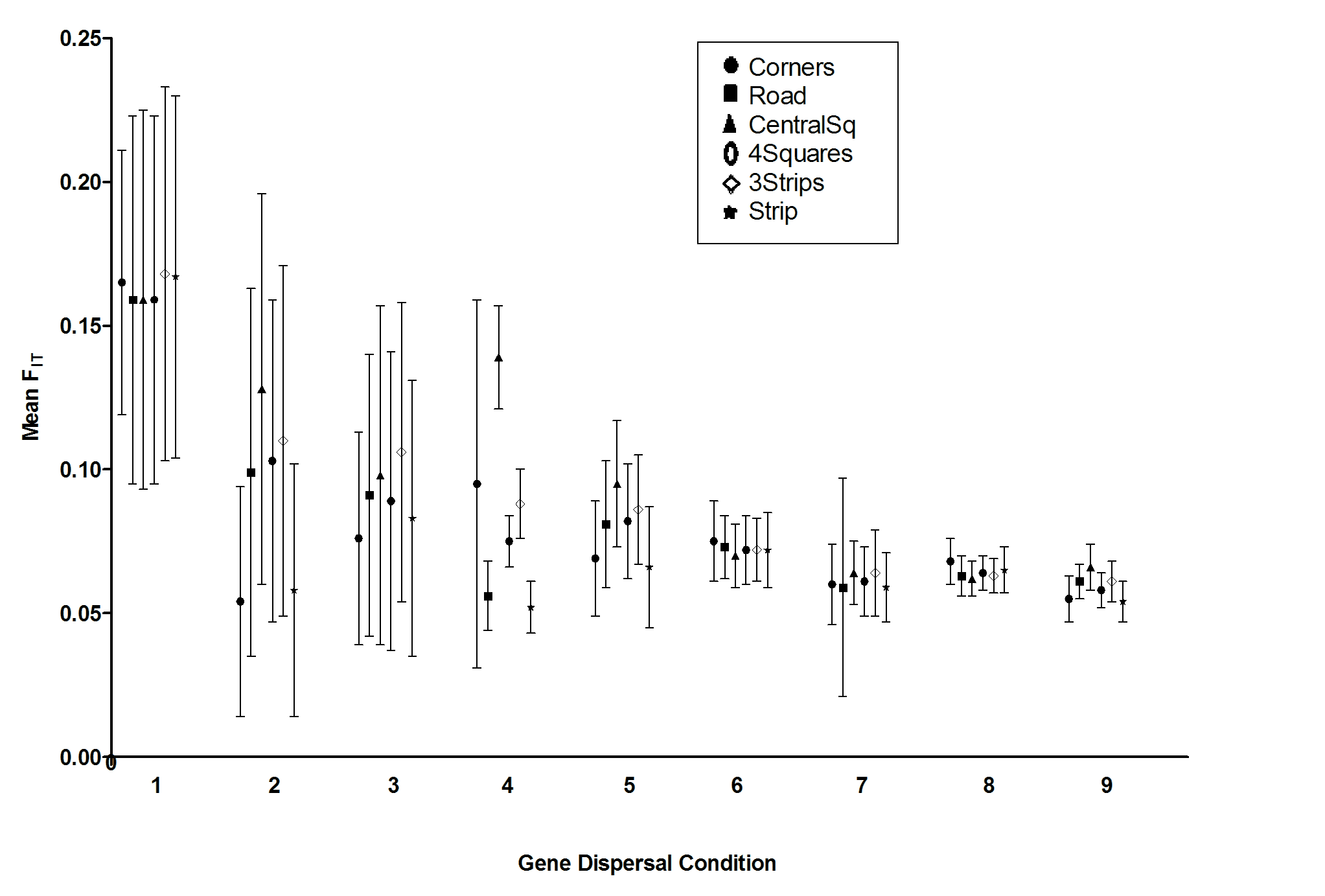

Supplement: S6 Fig — Trials varied in spatial logging patterns and gene dispersal distance (offspring and pollen). Groups labelled 1–9 indicate trials grouped according to gene dispersal condition in descending order of mean population: Gene dispersal conditions are abbreviated as follows, with spatial logging pattern in the legend appearing from left to right within each gene dispersal scenario: ‘N’ = Near, ‘E’ = Equal, ‘F’ = Far, ‘P’ = Pollen, ‘O’ = Offspring, ‘Equal’, ‘Near’ and ‘Far’ refer to the probability of offspring and/or pollen being dispersed to or being received from a particular distance frame relative to a pistillate individual (S1 Fig and S4 Table); 1 = FONP, 2 = FOFP, 3 = FOEP, 4 = NOFP, 5 = EOFP, 6 = EONP, 7 = EOEP, 8 = NONP, 9 = NOEP. “Control” refers to un-logged fragment, and “Selective Logging” refers to 10% removal of only those individuals belonging to the highest age classes. (TIF) [file pone.0127745.s008.tif]
