# Supplementary material for: Variable Gene Dispersal Conditions and Spatial Deforestation Patterns Can Interact to Affect Tropical Tree Conservation Outcomes
Source: PLoS One. 2015 May 22;10(5):e0127745. doi: 10.1371/journal.pone.0127745 (PMC4441416; doi:10.1371/journal.pone.0127745)
Supplement: S1 Table — (DOCX) [file pone.0127745.s009.docx]

**S1 Table. Similarities and differences between NEWGARDEN (NG) and other forest growth models including stand models, process-based models, empirical models, gap models and hybrid models.**

| **Similarities between NEWGARDEN (NG) and other forest growth models: stand models, process-based models, empirical models, gap models and hybrid models** | **Differences between NEWGARDEN (NG) and other forest growth models: stand models, process-based models, empirical models, gap models and hybrid models** |
| --- | --- |
| 1. Individual-based (like some process-based, gap and hybrid models) [45, 47, 51, 52, 53]. | 1. NG is not process-based (Physiological processes such as photosynthesis is not explicitly modeled). [45, 47]. |
| 2. Spatially-explicit (similar to later gap models where location is explicitly modeled). [45, 47, 52]. | 2. NG does not have population growth specified by a growth equation. [45, 47]. |
| 3. Seeks to predict short and long-term dynamics of the forest in response (e.g. population growth) to different biotic or abiotic variables. [45, 47, 51, 52, 53]. | 3. NG is driven by explicit life-history events which are specified by age-specific probabilities, e.g., mating, mortality, offspring and pollen dispersal. [45, 47]. |
| 4. Has a method to describe reproduction, dispersal, recruitment, establishment, growth and mortality. [45, 47, 51, 52, 53]. | 4. Spatial interactions are explicitly modeled by NG. E.g., offspring and pollen dispersal, reproduction, immigration. [45, 47]. |
|  | 5. NG currently models single species. [45, 47]. |
|  | 6. NG has no direct parameters for modeling abiotic variables such as climate, but the effects of these on life-history traits like age-specific reproduction, gene dispersal genetic diversity, and mortality can be modeled. [45, 47]. |
|  | 7. NG can model gap and non-gap situations. [45, 47]. |
|  | 8. NG is capable of high levels of replication of computer simulations allowing for robust statistical analyses. [45, 47]. |
|  | 9. NG explicitly calculates and monitors population growth, genetic diversity levels, and population genetic statistics such as F_st_ and F_it_ of the population through time. [45, 47]. |
|  | 10. NG can model spatial and temporal heterogeneity in the geography of the preserve being modeled. [45, 47]. |
|  | 11. NG can model a range of genetic histories such as presence of self- incompatibility alleles and inbreeding depression in the founders. [45, 47]. |
|  | 12. NG can model different mating systems ranging from bisexual to dioecious, as well as agamospermy and different levels of selfing. [45, 47]. |
