# Supplementary material for: Variable Gene Dispersal Conditions and Spatial Deforestation Patterns Can Interact to Affect Tropical Tree Conservation Outcomes
Source: PLoS One. 2015 May 22;10(5):e0127745. doi: 10.1371/journal.pone.0127745 (PMC4441416; doi:10.1371/journal.pone.0127745)
Supplement: S2 Table — (DOCX) [file pone.0127745.s010.docx]

**S2 Table.** **Age-specific rates of reproduction and pollen provisioning for the control equilibrium population.**

| **Age** | **Offspring production rate (r)** | **Pollen production** |
| --- | --- | --- |
| 0 | 0.00 | 0.00 |
| 139 | 0.00 | 0.00 |
| 140 | 0.50 | 0.10 |
| 168 | 2.50 | 0.15 |
| 196 | 7.80 | 0.20 |
| 216 | 7.80 | 0.60 |
| 236 | 8.00 | 1.00 |
| 268 | 8.00 | 1.00 |
| 295 | 8.00 | 1.00 |
| 365 | 8.00 | 1.00 |
| 396 | 8.00 | 1.00 |
| 425 | 6.00 | 0.75 |
| 426 | 0.00 | 0.00 |
