# Supplementary material for: Variable Gene Dispersal Conditions and Spatial Deforestation Patterns Can Interact to Affect Tropical Tree Conservation Outcomes
Source: PLoS One. 2015 May 22;10(5):e0127745. doi: 10.1371/journal.pone.0127745 (PMC4441416; doi:10.1371/journal.pone.0127745)
Supplement: S3 Table — (DOCX) [file pone.0127745.s011.docx]

**S3 Table.** **Age-specific mortality rate for the control equilibrium population**

| **Age** | **Mortality Rate** |
| --- | --- |
| 0 | 0.25 |
| 6 | 0.25 |
| 11 | 0.14 |
| 26 | 0.0075 |
| 51 | 0.0085 |
| 76 | 0.0095 |
| 101 | 0.01 |
| 151 | 0.01 |
| 201 | 0.01 |
| 251 | 0.01 |
| 301 | 0.01 |
| 401 | 0.01 |
| 425 | 0.01 |
| 426 | 0.01 |
