# Supplementary material for: Variable Gene Dispersal Conditions and Spatial Deforestation Patterns Can Interact to Affect Tropical Tree Conservation Outcomes
Source: PLoS One. 2015 May 22;10(5):e0127745. doi: 10.1371/journal.pone.0127745 (PMC4441416; doi:10.1371/journal.pone.0127745)
Supplement: S4 Table — Distance frame dimensions and selection probabilities for offspring and pollen dispersal distance from or to (respectively) an offspring-producing individual in a given mating event for the “Equal”, “Near” and “Far” gene dispersal categories. (DOCX) [file pone.0127745.s012.docx]

**S4 Table.** **Offspring and pollen dispersal distance dimensions.** Distance frame dimensions and selection probabilities for offspring and pollen dispersal distance from or to (respectively) an offspring-producing individual in a given mating event for the “Equal”, “Near” and “Far” gene dispersal categories.

| **Distance frame** | **Distance frame dimensions in x or y direction in grid points (gp) and maximum meters (m)** | **Probability of dispersing offspring for ‘Equal Offspring’ and receiving pollen for ‘Equal Pollen’.** | **Probability of dispersing offspring for ‘Near Offspring’ and receiving pollen for ‘Near Pollen’.** | **Probability of dispersing offspring for ‘Far Offspring’ and receiving pollen for ‘Far Pollen’.** |
| --- | --- | --- | --- | --- |
| a | 0-32 gp or 448 m | 0.20 | 0.80 | 0.05 |
| b | 32 – 64 gp or 896 m | 0.20 | 0.05 | 0.05 |
| c | 64 – 96 gp or 1,344 m | 0.20 | 0.05 | 0.05 |
| d | 96 -128 gp or 1,792 m | 0.20 | 0.05 | 0.05 |
| e | 128 – 160 gp or 2,240 m | 0.20 | 0.05 | 0.80 |
